# Strain Adjustment Realizes the Photocatalytic Overall Water Splitting on Tetragonal Zircon BiVO_4_

**DOI:** 10.1002/advs.202105299

**Published:** 2022-03-22

**Authors:** Dujuan Dai, Xizhuang Liang, Beibei Zhang, Yuanyuan Wang, Qian Wu, Xiaolei Bao, Zeyan Wang, Zhaoke Zheng, Hefeng Cheng, Ying Dai, Baibiao Huang, Peng Wang

**Affiliations:** ^1^ State Key Laboratory of Crystal Materials Shandong University Jinan 250100 China; ^2^ Institute of Fundamental and Frontier Sciences University of Electronic Science and Technology of China Chengdu 610000 China; ^3^ School of Physics Shandong University Jinan 250100 China

**Keywords:** charge separation, overall water splitting, strain engineering, tetragonal zircon BiVO_4_, visible light photocatalyst

## Abstract

Overall water splitting to generate H_2_ and O_2_ is vital in solving energy problem. It is still a great challenge to seek efficient visible light photocatalyst to realize overall water splitting. In this work, the tetragonal zircon BiVO_4_ is prepared by epitaxial growth on FTO substrate and its overall water splitting reaction is studied. Under the influence of epitaxial strain, the conduction band position shifts negatively and beyond H^+^/H_2_ reduction potential (0 V vs NHE), which enables it to possess the photocatalytic hydrogen evolution activity. After loading cocatalysts, the overall water splitting (*λ* > 400 nm) is realized (H_2_: ≈65.7 µmol g^−1^ h^−1^, O_2_: ≈32.6 µmol g^−1^ h^−1^), and the value of solar hydrogen conversion efficiency is 0.012%. The single‐particle photoluminescence (PL) spectra and PL decay kinetics tests demonstrate the cocatalysts are beneficial to the separation and transfer of carriers. The new strategy of adjusting the band structure by strain is provided.

## Graphical abstract

Under the influence of epitaxial strain, the conduction band position of tetragonal zircon BiVO_4_ grown on FTO substrate can across the H^+^/H_2_ reduction potential (0 V vs NHE), achieving overall water splitting under visible light. This work provides a new strategy of adjusting the band structure by strain for performing photocatalytic overall water splitting on BiVO_4_.

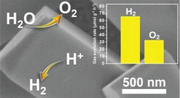

## 1 Introduction

In recent years, solar‐driven water splitting to produce clean hydrogen has been considered as a promising approach to solve energy and environmental issues. Since Honda and Fujishima used TiO_2_ to produce hydrogen and oxygen in 1972, photocatalytic water splitting has been one of the research hotspots.^[^ ^1^, ^2^, ^3^, ^4^, ^5^, ^6^, ^7^, ^8^, ^9^ ^]^ Recently, Domen's group used Al‐doped SrTiO_3_ to achieve a 96% external quantum efficiency of ultraviolet light from 350 to 360 nm,^[^ ^10^ ^]^ which led the development of photocatalysis, and ignited the hope of light‐driven water splitting. Although recent research on overall water splitting makes great progress,^[^ ^10^, ^11^, ^12^ ^]^ developing the efficient photocatalysts which have suitable band structure, visible light response, and high charge separation efficiency is still a great challenge.

BiVO_4_ as a chemically stable and visible‐light responsive photocatalyst have drawn much attention. It has three main crystal structures containing monoclinic scheelite (s‐m), tetragonal zircon (z‐t), and tetragonal scheelite (s‐t). Although the z‐t BiVO_4_ shows lower oxygen evolution activity than s‐m BiVO_4_, it does not prevent scientists from further exploring it. Our group has previously reported that z‐t BiVO_4_ can be used as photocathode to construct a bias‐free PEC cell.^[^ ^13^, ^14^ ^]^ In addition, no matter which crystal phase of BiVO_4_, as the conduction band minimum (CBM) is more positive than H^+^/H_2_ reduction potential, it has basic limitation of releasing H_2_. The current research on BiVO_4_ overall water splitting is mainly focused on constructing Z‐scheme systems and phase transition‐induced band edge engineering.^[^ ^15^, ^16^ ^]^ However, overall water splitting on pure z‐t BiVO_4_ still has not come true.

As we all know, the different lattice parameters of substrate and epitaxial layer may cause lattice mismatch, which leads to epitaxial growth with strain accumulation. The mismatch strain at interface will adjust its physical and chemical properties.^[^ ^17^, ^18^, ^19^, ^20^, ^21^ ^]^ Zheng et al. have reported the textured substrate accumulating strain locally greatly enhances the light absorption and surface reaction of the BiVO_4_ photoanode, reducing the total amount of light absorber required.^[^ ^22^ ^]^ Minseok Choi theoretically reported the tensile strain makes the CBM energy in s‐m BiVO_4_ very close to H^+^/H_2_ level, while the CBM energy in z‐t BiVO_4_ shifts upward or even higher than this level.^[^ ^23^ ^]^ However, there is still lack of general understanding of how epitaxial strain experimentally affects photocatalytic property of BiVO_4_. Here, we take z‐t BiVO_4_ as the research object, using unstrained BiVO_4_ powder and epitaxially strained BiVO_4_ to study the effect of strain on the band structure and overall water splitting.

## 2 Results and Discussion

### 2.1 Fabrication and Characterization of Tetragonal BiVO_4_

**Figure 1:**
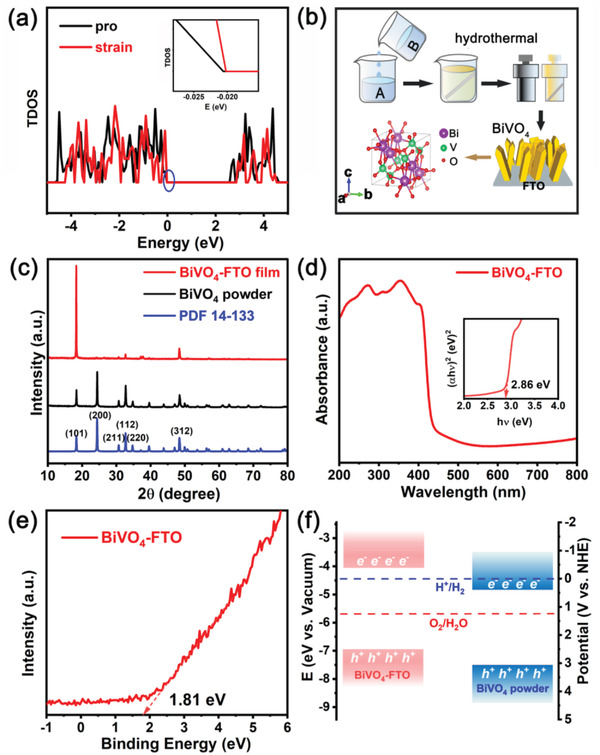
a) The band structure change induced by tensile strain on tetragonal BiVO_4_ based on DFT. b) Detailed preparation process for BiVO_4_‐FTO film and crystal structure of tetragonal BiVO_4_. c) XRD patterns of BiVO_4_‐FTO film and BiVO_4_ powder. d) UV–vis DRS spectrum and e) the energy level difference between the E_VB_ and Fermi level of BiVO_4_‐FTO sample. f) The band structure diagram of BiVO_4_‐FTO sample and BiVO_4_ powder.

When BiVO_4_ grows on another crystalline material, due to the different lattice parameters of two materials, strain will generate during the growth process. Specifically, when epitaxially growing on a substrate with larger lattice parameters, the BiVO_4_ unit cell (*a* = *b* = 0.730 nm) will suffer in‐plane tensile strain. The greater the lattice mismatch is, the larger the in‐plane tensile strain, leading to the change of each BiVO_4_ unit cell's lattice parameters (*a* = *b*).^[^ ^24^ ^]^ Based on the density functional theory (DFT), the influence of tensile strain on energy band structure of tetragonal BiVO_4_ is calculated. As shown in **Figure** **1**a, under tensile strain, the energy band of BiVO_4_ undergoes a certain degree of negative shift, in which the valence band (VB) moves to a small extent (inset of Figure 1a), and the conduction band (CB) moves significantly. So here we can envisage that under the effect of strain, the CB position of BiVO_4_ can be optimized, and stride the H^+^/H_2_ reduction potential. Combining with its intrinsic oxygen evolution activity, the z‐t BiVO_4_ has promise to achieve photocatalytic overall water splitting.^[^ ^15^, ^25^, ^26^ ^]^ Accordingly, the FTO glass consisting of tetragonal fluorine‐doped SnO_2_ (*a* = *b* = 0.476 nm) is chosen as a substrate (Figure S1, Supporting Information) which could subject the tetragonal BiVO_4_ film to the mismatch strain.^[^ ^27^, ^28^ ^]^ The uniform BiVO_4_ film is epitaxially growing on FTO substrate, and the detailed scheme of preparing process is depicted in Figure 1b. As shown, the tetragonal zircon BiVO_4_ unit cell consists of a regular VO_4_ tetrahedron (V—O bond length is 1.7062 Å) and a slightly twisted BiO_8_ dodecahedron (one Bi—O bond length is 2.4142 Å and another is 2.5489 Å, respectively). To make convenience for characterization and testing, the BiVO_4_ film grown on FTO substrate is scraped for collection, which is denoted as BiVO_4_‐FTO sample. In contrast, the naturally nucleated BiVO_4_ is prepared by coprecipitation method, hereinafter referred to BiVO_4_ powder. As shown in Figure 1c, all diffraction peaks are consistent with z‐t BiVO_4_ (space group I41/amd, JCPDS: 14‐133). Due to the same symmetry with the [101] direction of SnO_2_, the epitaxially grown BiVO_4_ shows strong crystallinity along the [101] orientation. The Raman and X‐ray photoelectron spectroscopy (XPS) spectra further show the z‐t BiVO_4_ is synthesized (Figures S2 and S3, Supporting Information).^[^ ^29^ ^]^ According to the diffuse reflectance spectrum (DRS) of BiVO_4_ (Figure 1d), the bandgap of BiVO_4_‐FTO is 2.86 eV. As seen from the ultraviolet photoelectron spectroscopy (UPS) spectra (Figure 1e and Figure S4a, Supporting Information), it can be determined that the E_VB_ and E_CB_ of BiVO_4_‐FTO sample are 2.47 and −0.39 eV, respectively. The E_CB_ is more negative than H^+^ to H_2_ reduction potential (0 V vs NHE), and E_VB_ is more positive than H_2_O to O_2_ oxidation potential (1.23 V vs NHE), indicating the BiVO_4_‐FTO sample has the possibility of photocatalytic overall water splitting. The specific calculation process and band position analysis of BiVO_4_ powder were depicted in the DRS and UPS spectra in Figure S4 (Supporting Information). Further, as shown in Figure 1f, there is a great difference between BiVO_4_‐FTO sample and BiVO_4_ powder at the edge of energy bands. The band arrangement of BiVO_4_ explains why the BiVO_4_‐FTO sample and BiVO_4_ powder exhibit significantly different photocatalytic activities.

**Figure 2:**
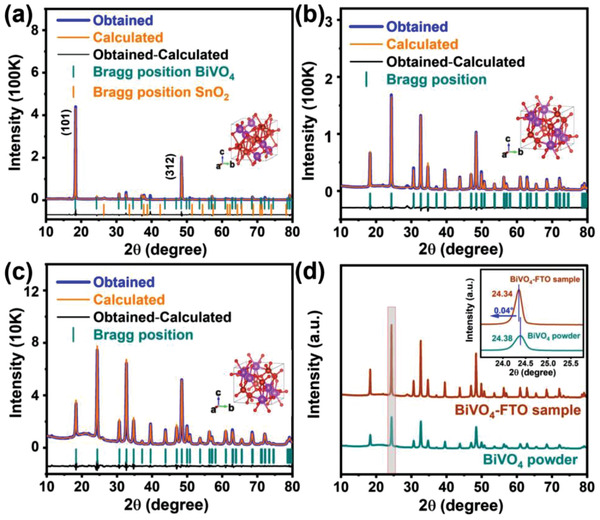
Rietveld‐refined XRD patterns of a) BiVO_4_‐FTO film, b) BiVO_4_‐FTO sample, and c) BiVO_4_ powder. d) XRD comparision of BiVO_4_‐FTO sample and BiVO_4_ powder. Inset: the enlarged image of main peak (200).

X‐ray diffraction method was used to prove the existence of residual strain. The residual stress can be calculated from the strain which is measured by XRD diffraction.^[^ ^30^ ^]^ According to the material parameters (Table S1, Supporting Information), the residual stress is 390.29 ± 47.44 MPa, as shown in Supporting Information 2. Although scratching will cause some stress loss, the increase in cell parameters caused by strain is irreversible, which also can be proved by XRD refinement. Through XRD data refinement (**Figure** 2a‐c), all the diffraction peaks of BiVO_4_‐FTO film and BiVO_4_‐FTO sample as well as BiVO_4_ powder are consistent with the Bragg position (represented by the blue‐green vertical line) of tetragonal zircon BiVO_4_ with space group I4_1_/amd (PDF: 14‐133). It is worth noting that compared with the naturally nucleated BiVO_4_ powder, the main peak of BiVO_4_‐FTO sample shifts toward lower angle (Figure 2d), which corresponds to the larger lattice parameters after tensile strain. The detailed crystal structures and unit cell parameters are further established (see Table S2, Supporting Information). As known, when BiVO_4_ grows on the substrate with larger lattice parameters, it will suffer in‐plane tensile strain and lattice parameters will change.^[^ ^24^ ^]^ Compared with BiVO_4_ powder (*a* = *b* = 7.3007 Å), the BiVO_4_‐FTO film and BiVO_4_‐FTO sample collected from the substrate have the same in‐plane lattice parameters (*a* = *b* = 7.3044 ≈ 7.3047 Å), which is larger than that of naturally nucleated BiVO_4_ powder.

**Figure 3:**
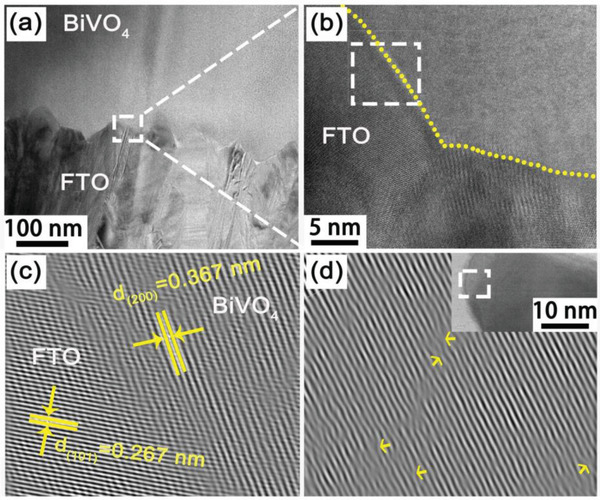
a) Cross‐sectional FIB‐TEM image of BiVO_4_‐FTO film coated by thin Pt conductive layer. b) FIB‐HRTEM image taken from the region of white square in (a). c) Fourier space filtered image of white square in (b). d) Fourier space filtered image taken from the region of white square in illustration (Inset: TEM image of BiVO_4_‐FTO sample closed to the FTO substrate).

**Figure** **3** depicts cross‐sectional FIB‐TEM images of BiVO_4_‐FTO. As shown in Figure 3a, continuous BiVO_4_ film grows on the surface of FTO, which can completely bridge the gap at the interface. Figure 3b is the enlarged view of selected area of Figure 3a. The yellow dotted line is the interface between FTO substrate and BiVO_4_ growth layer, and the contact interface is the region where a large number of lattice distortion occur. Therefore, the FIB‐HRTEM image in Figure 3c reveals the defect details of the selected white area in Figure 3b. The interplanar spacings of 0.367 and 0.267 nm are ascribed to (200) plane of BiVO_4_ and (101) plane of SnO_2_. Due to the incomplete matching of the interplanar spacing between SnO_2_ and BiVO_4_ in the [101] direction, BiVO_4_ bears strain from interface and shows deformation near the contact interface between them. The BiVO_4_ sample growing on FTO substrate was carefully scraped off and analyzed by HRTEM. After fast Fourier transform, it is found that there are still lattice distortions in the region near the FTO substrate (white square in the inset), which is consistent with the refined XRD results, indicating the interface strain exist between substrate and film during epitaxial growth progress (Figure 3d).

**Figure 4:**
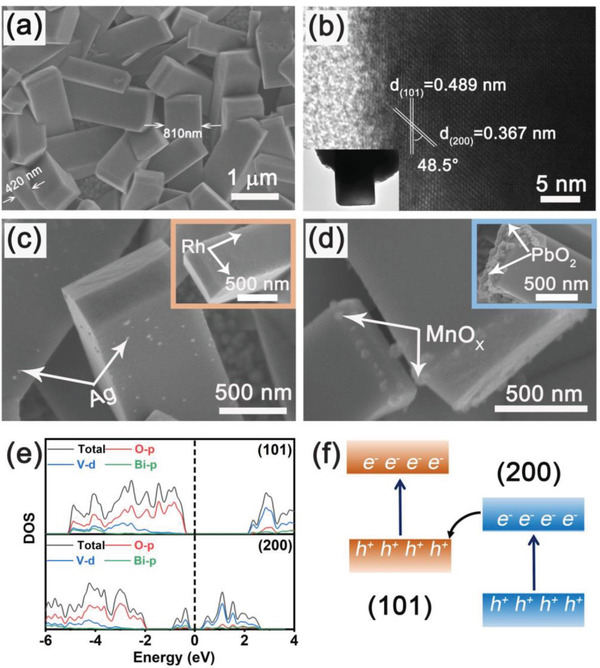
a) SEM image of BiVO_4_‐FTO film. b) HRTEM image of BiVO_4_‐FTO sample. c) SEM images of BiVO_4_‐FTO film with the photoreduction and d) photooxidation deposition. e) Density of states (DOS) of (200) and (101) planes of tetragonal BiVO_4_ at equilibrium. f) Schematic diagram of band structure between crystal planes and electron flow direction.

The scanning electron microscope (SEM) image of BiVO_4_‐FTO film nanocrystals is shown in **Figure** **4**a which presents the uniformly distributed cuboid with the width about 810 nm and thickness about 420 nm. More detailed structure of collected BiVO_4_‐FTO sample was further studied by HRTEM (Figure 4b). The crystalline lattice spacing of *d* = 0.489 and 0.367 nm can be attributed to the (101) and (200) planes of tetragonal BiVO_4_, and the included angle of 48.5° is consistent with the theoretical value of the angle between (101) and (200) planes. In contrast, according to the XRD (Figure 1c) and SEM (Figure S5, Supporting Information), no obvious orientation of the naturally nucleated BiVO_4_ powder was found. Due to different crystal planes have different redox ability, the inherent charge separation characteristics of BiVO_4_‐FTO photocatalyst were studied by photochemical deposition method. Through SEM imaging, the different migration of photogenerated carriers can be proven. The detailed results are shown in Figure 4c,d. Metals (Ag, Rh) are selectively deposited on the (101) planes, where the photogenerated electrons accumulate and reduce the metal ions (Ag^+^, Rh^3+^) to metals. Due to the metal (Ag, Rh) particles are small in Figure 4c, detailed SEM images of BiVO_4_‐FTO photocatalyst loaded with Ag or Rh are provided in Figure S6 (Supporting Information). Similarly, metal oxides (MnO_x_, PbO_2_) are selectively deposited on the (200) planes under light irradiation where photogenerated holes accumulate and oxidize metal ions (Mn^2+^, Pb^2+^) to metal oxides. The relative position of energy levels of different crystal planes can be evaluated by the density functional theory (DFT) calculation.^[^ ^31^, ^32^, ^33^ ^]^ According to the results in Figure 4e, compared with (200) crystal plane, the (101) crystal plane of BiVO_4_ shows the higher CBM and valence band maximum (VBM) position, and the VBM of (101) crystal plane is closer to the CBM of (200) crystal plane. This relative energy level position has a positive effect on the formation Z‐type arrangement. Reasonably, the SEM results of photodeposition can be further explained by the electron flow pattern in Figure 4f, where (101) planes tend to be electron enriched and (200) planes tend to be hole enriched.

### 2.2 Photocatalytic Activity of BiVO_4_

**Figure 5:**
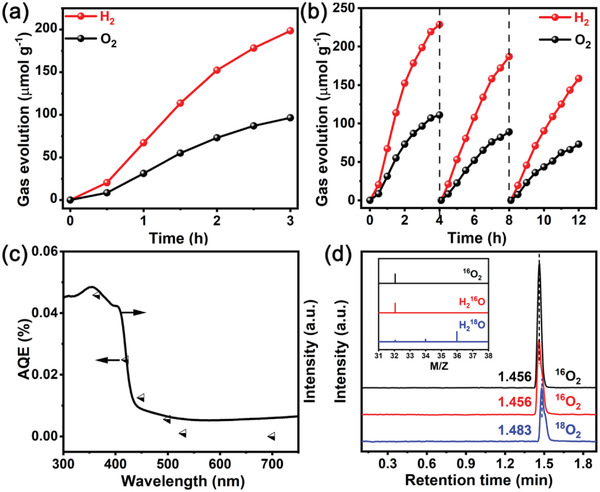
Photocatalytic performance of BiVO_4_‐FTO sample deposited with Rh/Cr_2_O_3_/MnO_x_. a) Photocatalytic overall water splitting reaction under visble light (*λ* > 400 nm). b) Time course of overall water splitting reaction. c) The wavelength‐dependent apparent quantum efficiency (AQE) and UV–vis diffuse reflectance spectrum. d) GC‐MS spectra and analysis over BiVO_4_‐FTO sample after irradiation for 6 h with different oxygen sources.

The light absorption of FTO was tested, which appears at about 350 nm (Figure S8, Supporting Information). To exclude its contribution to H_2_ production, the photocatalytic activities of tetragonal BiVO_4_ with and without epitaxial strain were tested under visible light irradiation (*λ* > 400 nm). As indicated in Figure S9 (Supporting Information), bare BiVO_4_ powder cannot release H_2_, while bare BiVO_4_‐FTO sample releases small amount of H_2_. Therefore, to ensure the reaction gas release smoothly, the dual cocatalysts Rh/Cr_2_O_3_ and MnO_x_^[^ ^10^, ^34^ ^]^ were loaded by photodeposition method. The loading of cocatalysts is proved by SEM, XPS, and EDS analysis (Figures S10–S12, Supporting Information). SEM images (Figure S10, Supporting Information) demonstrate the deposition of cocatalysts on specific crystal facets, which is consistent with SEM observations (Figure 4). Moreover, XPS and EDS results (Figures S11 and S12, Supporting Information) further confirm that the cocatalysts are successfully deposited on the surface of BiVO_4_. The LSV analysis of tetragonal BiVO_4_ before and after loading cocatalysts is shown in Figure S13 (Supporting Information). After loading dual cocatalysts, BiVO_4_‐Rh/Cr_2_O_3_/MnO_x_ yields higher photocurrent than pure BiVO_4_ and single cocatalyst BiVO_4_‐Rh/Cr_2_O_3_, which proves that more photogenerated carriers accumulate on the surface of BiVO_4_‐Rh/Cr_2_O_3_/MnO_x_ to participate in surface reactions. The half‐reaction of hydrogen evolution (HER) and oxygen evolution (OER) in the presence of sacrificial agents have been operated (Figure S14, Supporting Information). Compared with BiVO_4_ powder, BiVO_4_‐FTO sample shows higher photocatalytic HER and OER activity, which is caused by improved reduction ability and carriers’ separation efficiency. Further, because s‐m phase BiVO_4_ is usually used for photocatalytic water oxidation among the BiVO_4_ polymorphs, the photocatalytic property of s‐m BiVO_4_ is also studied for comparison (Figure S15, Supporting Information). Although s‐m BiVO_4_ has higher oxygen evolution activity in the presence of sacrificial agent, it cannot evolute H_2_ due to insufficient reduction ability. In addition, s‐m BiVO_4_ cannot epitaxial grow on FTO substrate because of the different crystal structure and too large lattice mismatch between s‐m BiVO_4_ and SnO_2_. As shown in **Figure** **5**a, under visible light irradiation (*λ* > 400 nm), the simultaneous release of hydrogen and oxygen is achieved on BiVO_4_‐FTO sample with cocatalysts, and the solar hydrogen conversion efficiency (STH) is 0.012%. Other single‐step overall water splitting photocatalysts are shown in Table S3 (Supporting Information). It can be clearly seen from Figure 5b that the water splitting activity of the photocatalyst gradually decreases during the 12 h irradiation period. The origin of this phenomenon was further studied. The XRD patterns before and after the reaction didn't change significantly, indicating the BiVO_4_‐FTO sample still retains the crystal structure (Figure S16, Supporting Information). However, the ICP‐OES measurement prove that vanadium ions are dissolved as the photocatalytic reaction proceeds (Figure S17, Supporting Information), indicating the decrease in stability is mainly due to the photoinduced dissolution of V^5+^ ions, leading to the severe photocorrosion, which is consistent with the previously report.^[^ ^35^, ^36^ ^]^ The stability of BiVO_4_ has been a hot and challenging issue for a long time, further exploration of strategies to improve the photostability of tetragonal BiVO_4_ is the focus of our follow‐up research. The wavelength‐dependent apparent quantum efficiency (AQE) of BiVO_4_‐FTO sample is depicted in Figure 5c. The AQE values match well with the diffuse reflectance spectrum, demonstrating that the overall water splitting is caused by the absorption of incident light. To determine the oxygen source of the reaction product, gas chromatography‐mass spectrometer (GC‐MS) test was applied to detect ^16^O_2_ and ^18^O_2_. As shown in Figure 5d, the retention time of ^16^O_2_ and ^16^O_2_ from H_2_^16^O are earlier than that of ^18^O_2_ from H_2_^18^O. GC‐MS analysis (shown in the inset of Figure 5d) further shows that the main product with H_2_^16^O as the oxygen source is ^16^O_2_ (m/z = 32), while the main product with H_2_^18^O as the oxygen source is ^18^O_2_ (m/z = 36). Therefore, it is clear that the photocatalytic reaction product originates from the H_2_O splitting. To verify the effect of the crystal planes on photocatalytic hydrogen production, strong alkali was used to treat BiVO_4_‐FTO film. It can be seen from Figure S18 (Supporting Information) that the etched BiVO_4_‐FTO retain the crystal structure and chemical composition basically but the original smooth crystal planes are corroded (Figure S19, Supporting Information). The etched sample are collected for photocatalytic hydrogen test and the activity is greatly reduced (Figure S20, Supporting Information), revealing that crystal planes are beneficial to the separation of carriers. Moreover, the tetragonal BiVO_4_‐FTO film with different redox crystal facets exposure ratio are provided in Figure S21 (Supporting Information), which are obtained by adjusting the pH of precursor. By comparing the water splitting activity in Figure S22 (Supporting Information), it is found that under the same loading of cocatalysts, the activity of BiVO_4_‐FTO (pH = 2) is higher than those of BiVO_4_‐FTO (pH = 1.6) and BiVO_4_‐FTO (pH = 3), indicating that the properly exposed redox crystal facets are beneficial to the photocatalytic water splitting reaction. Surface photovoltage (SPV) measurements (Figure S23, Supporting Information) indicate that BiVO_4_‐FTO sample has higher photovoltage than BiVO_4_ powder, confirming the effective separation and transportation of photogenerated carriers in BiVO_4_‐FTO sample. In addition, BiVO_4_‐FTO film with different thicknesses can be obtained by adjusting the hydrothermal time (Figures S24 and S25, Supporting Information). It can be seen that as the synthesis time increases from 3 to 24 h, the hydrogen evolution rate gradually decreases (Figure S26a, Supporting Information), which is associated with the reduction of lattice strain. In addition, after further loading the cocatalysts, the gas evolution rates are significantly increased (Figure S26b, Supporting Information), and the best gas release rate is achieved at the synthesis time of 12 h. Furthermore, to prevent the presence of incompletely consumed organic reactants in the product from affecting the source of hydrogen, infrared analysis is carried out, and no obvious organic peaks of reactant is detected (Figure S27a, Supporting Information). Moreover, it can be seen from the TG analysis (Figure S27b, Supporting Information) that from ambient temperature to 600 °C, the BiVO_4_‐FTO sample shows basically no change when heated in air atmosphere. The slight weight loss of BiVO_4_ powder can be attributed to the evaporation of adsorbed water on the surface. The above results indicate that the tetragonal BiVO_4_ has good thermodynamic stability.

**Figure 6:**
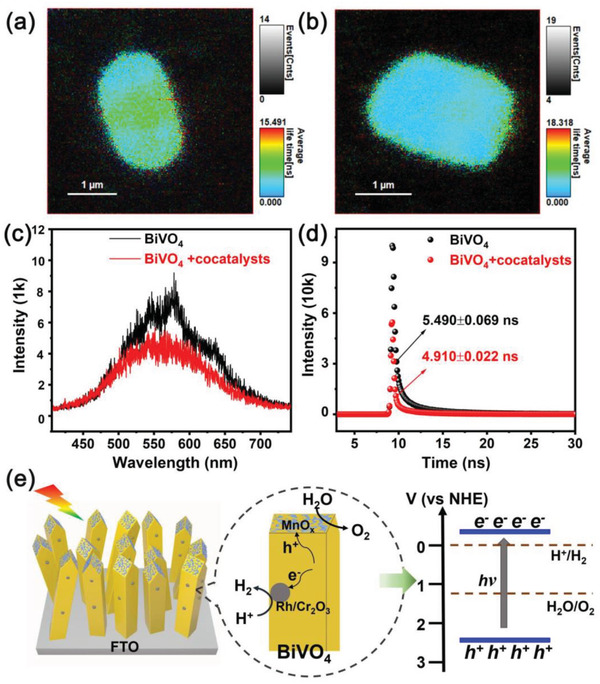
PL images of single a) BiVO_4_‐FTO sample and b) BiVO_4_‐FTO sample with cocatalysts. c) PL intensities and d) PL decay spectra of single BiVO_4_‐FTO sample with and without cocatalysts. e) Schematic diagram of photocatalytic overall water splitting on tetragonal zircon BiVO_4_ loaded with Rh/Cr_2_O_3_/MnO_x_ cocatalysts.

Here, the single‐particle photoluminescence (PL) spectra and PL decay kinetics tests were used to explore the role of cocatalysts in photocatalytic overall water splitting reaction. Compared with the ordinary photoluminescence spectra, the single particle photoluminescence has less noise and interference from other molecules or particles, so the role of cocatalyst can be studied from the perspective of single particle. PL images of single BiVO_4_‐FTO sample and BiVO_4_‐FTO sample with cocatalysts are depicted in **Figure** **6**a,b. Compared with Figure 6a, the blue area in the center of Figure 6b represents the shorter average life time, which means that after loading cocatalysts, the photogenerated carriers are quickly captured by the cocatalysts and participate in the water splitting reaction. As shown in Figure 6c, the significant decreased intensity of PL peak is observed, indicating the reduced recombination of photogenerated carriers on BiVO_4_‐FTO sample with cocatalysts. Further, according to the three‐exponential fitting (*a*_1_·exp(*τ*/*τ*_1_) + *a*_2_·exp(*τ*/*τ*_2_) + *a*_3_·exp(*τ*/*τ*_3_)) shown in Figure 6d, it is determined that the lifetime of BiVO_4_‐FTO sample with cocatalysts (≈4.910 ± 0.022 ns) is shorter than that of BiVO_4_‐FTO sample (≈5.490 ± 0.069 ns), demonstrating the carriers are separated and transferred more quickly after loading cocatalysts.^[^ ^37^ ^]^ The schematic diagram of photocatalytic overall water splitting on tetragonal zircon BiVO_4_ loaded with cocatalysts is shown in Figure 6e. Under the visible‐light excitation, photogenerated electrons and holes are generated at the CBM and VBM of tetragonal BiVO_4_, which then migrate to the H_2_‐evolution cocatalyst Rh/Cr_2_O_3_ and O_2_‐evolution cocatalyst MnO_x_ to participate the surface redox reaction, respectively. This spatially separated structure is beneficial to inhibit the recombination of photogenerated carriers and promote photocatalytic overall water splitting reaction.

## 3 Conclusion

In summary, tetragonal BiVO_4_ on FTO substrate was prepared and overall water splitting under visible light irradiation was evaluated. The BiVO_4_ grown on substrate has better catalytic performance than the naturally nucleated BiVO_4_. By introducing strain, the energy band position could be effectively adjusted, so that conduction band position shifts up and exceeds the hydrogen evolution potential. The common exposure of oxidizing crystal planes and reducing crystal planes increase the separation efficiency of photogenerated carriers and promote photocatalytic reaction. Considering the s‐m BiVO_4_ has better light absorption and oxygen generation kinetics, the works on monoclinic BiVO_4_ and other substrate are in procedure. The new initiative is put forward to adjust the band structure of photocatalyst for overall water splitting under visible light irradiation.

## 4 Experimental Section

### Preparation of BiVO_4_‐FTO Film

All chemicals are of analytical grade and do not require further purification when used. The BiVO_4_‐FTO film was grown by hydrothermal method. Typically, the precursor Bi (NO_3_)_3_·5 H_2_O (1 mmol) was dissolved in HNO_3_ solution (20 mL, 2 m), and C_10_H_14_N_2_Na_2_O_8_ (1 mmol) was added and stirred for 30 min to obtain solution A. At the same time, NH_4_VO_3_ (1 mmol) was dissolved in NaOH solution (40 mL, 1 m), and then C_10_H_14_N_2_Na_2_O_8_ (1 mmol) was added and stirred for 30 min to obtain solution B. Then A solution was slowly poured into the B solution and the solution became a clear yellow solution. After adjusting the pH to 2, continue to stir the solution for another 30 min and transfer to 100 mL Teflon‐lined autoclave. The FTO glass was sonicated sequentially in acetone, ethanol, and isopropanol for 30 min. Then, put the cleaned FTO glass (3 cm × 4 cm) into 100 mL Teflon‐lined autoclave at an angle, with the conductive surface facing down. After sealing the autoclave, the hydrothermal reaction was carried out at 160 °C for 12 h. The autoclave was naturally cooled to room temperature, and then the FTO glass was taken out, rinsed with deionized water several times, and dried at room temperature. The obtained product was called BiVO_4_‐FTO film. To make the test convenience, the scraped sample was called BiVO_4_‐FTO sample.

### Surface Selective Photodeposition

To perform photooxidation deposition, the BiVO_4_‐FTO film was immersed in an aqueous solution containing 0.2 × 10^−3^ m KIO_3_ (100 mL), 0.1 × 10^−3^ m Mn (NO_3_)_2_ for MnO_x_ deposition, and 0.1 × 10^−3^ m Pb (NO_3_)_2_ for PbO_2_ deposition, respectively. The reaction system was irradiated under a 300 W Xe lamp (full arc) for 10 min. After irradiation, the BiVO_4_‐FTO film was washed with deionized water and ethanol and dried. For photoreduction deposition, the steps were almost the same, except that the deposition aqueous solution was composed of 10% (volume ratio) methanol, 0.1 × 10^−3^ m AgNO_3_ for Ag deposition and 2 mg mL^–1^ RhCl_3_·3H_2_O for Rh deposition, respectively.

### Preparation of BiVO_4_ Powder

BiVO_4_ powder was synthesized by coprecipitation method. Typically, Bi (NO_3_)_3_·5H_2_O (2 mmol) and NH_4_VO_3_ (2 mmol) were simultaneously dissolved in 60 mL of deionized water and stirred vigorously for 2 h. The reaction product was filtered, washed with deionized water and ethanol, and dried in an oven at 100 °C for 5 h. The obtained product was called BiVO_4_ powder.

### Computational Details

All the calculations were performed by using the Vienna Ab Initio Simulation Package (VASP).^[^ ^38^ ^]^ The ion–electron interactions were described by projector augmented wave (PAW) method.^[^ ^39^ ^]^ The generalized gradient approximation (GGA) in the Perdew–Burke–Ernzerh (PBE) with a cutoff energy of 500 eV for exchange–correlation interactions were adopted.^[^ ^40^ ^]^ A Monkhorst–Pack 7 × 7 × 1 k‐point grid was adopted for all the calculations. The convergence criteria for the force and energy were set to be 0.01 eV Å^−1^ and 10^−4^ eV with a vacuum space larger than 20 Å in the *z*‐direction to avoid interactions between periodic units during the structure relaxation. The magnitude of strain is described by *a*/*a*_0_, here *a*_0_ and *a* denote the lattice parameters of the unstrained and strained systems, respectively. As a result, 10% in‐plane tensile strain is applied to (101) plane of BiVO_4_.

### Characterization

XRD patterns were conducted on a Bruker AXS D8 diffractometer equipped with Cu K*α* radiation to reveal the crystal structure. Morphologies were investigated by SEM (Hitachi S‐4800) equipped with an EDS. The UV–vis DRS analyses were recorded by Shimadzu UV‐2550 spectrophotometer using BaSO_4_ as reflectance standard to explore the optical absorption. The TEM and HRTEM tests were performed with a JEOL JEM‐2100F microscope to analyze the nanostructure and composition of the as‐prepared BiVO_4_ photocatalyst. The cross‐sectional FIB‐HRTEM samples were analyzed by focused ion beam (FIB) technology. UPS measurements of the as‐prepared samples were performed with He I (21.2 eV) as monochromatic light source and a total instrumental energy resolution of 100 meV. XPS measurements were carried out on a Thermo ESCALAB 250XI spectrometer with a monochromatic Al‐K*α* source to explore the element composition and valence states on the surface, and the binding energies were calibrated by the C 1s peak (284.8 eV). Raman spectra were measured on LabRAM HR800 with laser excitation of 532 nm. The reaction liquid used for gas measurement was analyzed by ICP‐OES/AES: Varian (720‐ES). The amounts of H_2_ and O_2_ evolution were analyzed using gas chromatography (GC‐7920) equipped with a thermal conductivity detector (Ar as carrier gas). Single particle fluorescence and fluorescence decay measurements were carried out on a confocal microscope instrument. A 375 nm continuous wave (CW) laser was used to excite the interband transition of BiVO_4_ and pulse frequency was 5 MHz. To detect the source of oxygen, GCMS‐QP2010 was used to detect oxygen isotope. The software used for XRD data refinement is TOPAS academic.

### Photocatalytic Reactions

The photocatalytic reactions were carried out in a Pyrex reaction cell connected to a closed gas circulation and evacuation system. Typically, the area of the BiVO_4_‐FTO film is 12 cm^2^, on which the mass is estimated to be ≈5 mg. Generally, 10 mg photocatalyst was immersed in 100 mL of reaction solution. The photocatalytic HER, OER, and overall water splitting reaction were carried out in 0.02 × 10^−3^ m H_2_O_2_, 0.02 m AgNO_3_ and pure water, respectively. The theoretical loading amounts were 2, 2, and 0.5 wt% for Rh, Cr, and MnO_x_. For the overall water splitting reaction, after the catalyst was ultrasonically dissolved, the cocatalyst precursor was sequentially added to the reaction solution to carry out the photodeposition reaction. First, a specific amount of RhCl_3_·3H_2_O solution (2 mg mL^−1^) was added to the reaction system, and irradiated for 10 min. Subsequently, the K_2_CrO_4_ (2 mg mL^−1^) and Mn(NO_3_)_2_ (0.1 × 10^−3^ m) solution were added to the suspension and irradiated for another 5 min, respectively. The system was evacuated for 30 min to ensure complete removal of air, and then illuminated from the top surface with a 300 W Xe lamp (PLS‐SXE300D, Beijing PerfectLight Technology Co., Ltd.) equipped with a 400 nm cut‐off filter (*λ* > 400 nm). A cooling water stream was used to maintain the reaction suspension at 288 K. The separated gas was analyzed by gas chromatography (GC‐7290, TCD with Ar as a carrier gas). The photocatalytic stability test was performed every 4 h as a cycle. After each independent cycle, the photocatalyst was recycled by centrifugation and redispersed in new solution. The wavelength dependence of the AQE was tested under the same photocatalytic reaction conditions, through the 365, 420, 450, 500, 530, or 700 nm bandpass filters and the masked area of 1 cm^2^. The photon flux of the incident light was determined using a PL‐MW2000 spectrophotometer (PerfectLight, China). The AQE was calculated from the ratio of the number of electrons reacted to the number of incident photons during the water splitting process. The calculation formula is

(1) $$\mathrm{AQE}\;=\frac{2\times \text{the}\;\text{number}\;\text{of}\;\text{evolved}\;\text{hydrogen}\;\text{molecules}}{\text{the}\;\text{number}\;\text{of}\;\text{incident}\;\text{photons}}\;\times 100\%$$

The STH efficiency was measured under simulated sunlight (AM 1.5G, 1 cm^2^ irradiation area). The calculation formula is

(2) $$\mathrm{STH}\;=\frac{\text{output}\;\text{energy}\;\text{as}\;H_{2}}{\text{energy}\;\text{of}\;\text{incident}\;\text{solar}\;\text{light}}\;$$

## Conflict of Interest

The authors declare no conflict of interest.

## Supporting information

Supporting Information

Supporting Information

## Data Availability

The data that support the findings of this study are available from the corresponding author upon reasonable request.
