# Supplementary material for: Strain Adjustment Realizes the Photocatalytic Overall Water Splitting on Tetragonal Zircon BiVO4
Source: Adv Sci (Weinh). 2022 Mar 22;9(15):2105299. doi: 10.1002/advs.202105299 (PMC9131610; doi:10.1002/advs.202105299)
Supplement: Supplementary file 1 — Supporting Information [file ADVS-9-2105299-s002.pdf]

ADVANCED SCIENCE
Open Access

Supporting Information

for *Adv. Sci.*, DOI 10.1002/advs.202105299

Strain Adjustment Realizes the Photocatalytic Overall Water Splitting on Tetragonal Zircon $BiVO_4$

*Dujuan Dai, Xizhuang Liang, Beibei Zhang, Yuanyuan Wang, Qian Wu, Xiaolei Bao, Zeyan Wang, Zhaoke Zheng, Hefeng Cheng, Ying Dai, Baibiao Huang and Peng Wang**

Supporting Information

Strain Adjustment Realizes the Photocatalytic Overall Water Splitting on Tetragonal Zircon $BiVO_4$

*Dujuan Dai, Xizhuang Liang, Beibei Zhang, Yuanyuan Wang, Qian Wu, Xiaolei Bao, Zeyan Wang, Zhaoke Zheng, Hefeng Cheng, Ying Dai, Baibiao Huang, and Peng Wang**

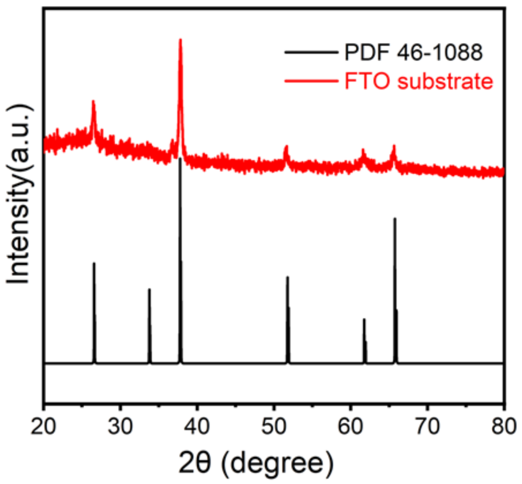

Figure S1 XRD pattern of FTO substrate.

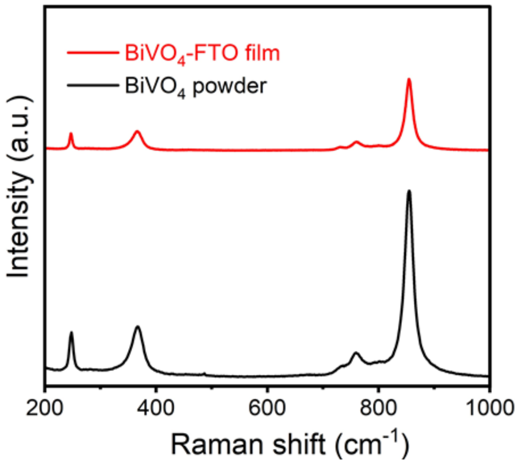

Figure S2 Raman spectra of $BiVO_4$-FTO film and $BiVO_4$ powder.

The Raman spectra show two bands at 760 and 855 cm^{-1}, which can be attributed to the antisymmetric V-O stretching mode υ_{as} (V-O) and the symmetric V-O stretching mode υ_s (V-O) of tetragonal zircon $BiVO_4$. In the 200-400 cm^{-1} region with two bands at 365 and 247 cm^{-1}, which are assigned to the VO_4 bending mode δ_s (VO_4) and external vibration mode.[1]

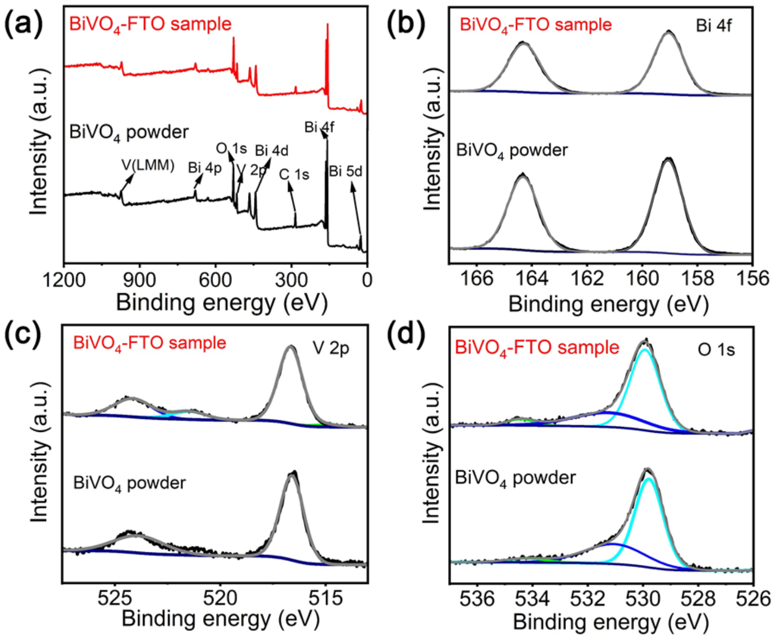

Figure S3 (a-d) XPS spectra of $BiVO_4$-FTO sample and $BiVO_4$ powder.

Figure S3 shows the XPS spectra of $BiVO_4$-FTO sample and $BiVO_4$ powder, and **Figure S3a** displays the XPS survey spectra. The Bi 4f spectra are shown in **Figure S3b**, where Bi $4f_{7/2}$ and Bi $4f_{5/2}$ peaks at 159.03 and 164.34 eV respectively, indicating the presence of Bi^{3+} in $BiVO_4$. V $2p_{1/2}$ and V $2p_{3/2}$ characteristic peaks which located at 524.13 eV and 516.69 eV (**Figure S3c**) indicates the presence of V^{5+} in $BiVO_4$. For the $BiVO_4$-FTO sample, the peaks at 521.36 eV and 514.9 eV are assigned to V $2p_{3/2}$ and V $2p_{1/2}$ of V^{4+}, respectively. V^{5+} is partly self-reducing to V^{4+}, indicating the $BiVO_4$-FTO sample is beneficial to the accumulation of electrons, so as to better carry out the reduction reaction.[2] Three peaks were detected in the O 1s region (**Figure S3d**): the peak at 529.9 eV can be assigned to the lattice oxygen in $BiVO_4$, while the peaks at 531.31 eV and 534.37 eV are derived from the signal of oxygen vacancy and oxygen adsorbed on the sample surface.[3] Besides, the difference in the binding energy of O 1s between $BiVO_4$-FTO sample and $BiVO_4$ powder is negligible.

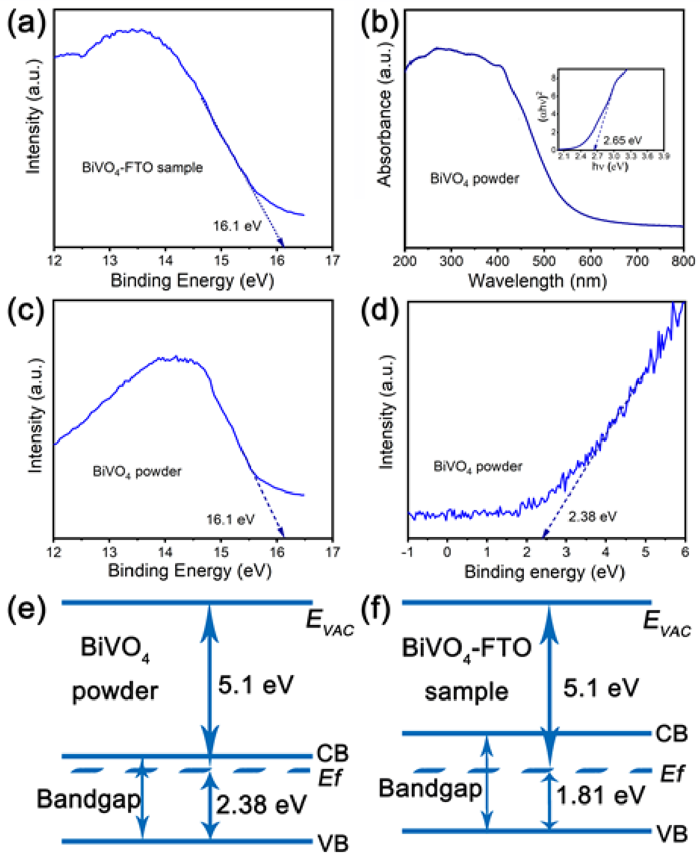

Figure S4 (a) The energy of the cutoff edge of secondary electron emission of $BiVO_4$-FTO sample. (b) UV-vis DRS spectrum of $BiVO_4$ powder. (c) The energy of the cutoff edge of secondary electron emission of $BiVO_4$ powder. (d) The energy level difference between the E_{VB} and Fermi level of $BiVO_4$ powder. Schematic diagram with band position of (e) $BiVO_4$ powder and (f) $BiVO_4$-FTO sample.

The UPS spectra were measured with a monochromatic He I light source (21.2 eV), and the work function (φ) can be obtained by the formula "$\varphi = h\upsilon - (E_{cutoff}-E_F^0)$", where hυ is the excitation energy of the monochromatic He I light source (21.2 eV), E_{cutoff} is the energy of the secondary electron emission cut-off edge, and E_F^0 is the Fermi level of the instrument after calibration with Au standard sample ($E_F^0 = 0$). According to E_{cutoff} (**Figure S4a,c**), the work functions of $BiVO_4$-FTO sample and $BiVO_4$ powder are both 5.1 eV. As shown in **Figure 1e**, the energy level difference between the E_{VB} and Fermi level (E_F) of $BiVO_4$-FTO sample is 1.81 eV, so E_{VB} is determined to be -6.91 eV (vs. vacuum level). According to the bandgap of $BiVO_4$-FTO (2.86 eV) shown in **Figure 1d**, the E_{CB} of the $BiVO_4$-FTO sample is -4.05 eV (vs. vacuum level). Taking 0 V (vs. NHE) equal to -4.44 eV (vs. vacuum level) as reference,[4] the E_{VB} and E_{CB} of $BiVO_4$-FTO sample are 2.47 eV and -0.39 eV (vs. NHE), respectively. Due to the energy level difference between the E_{VB} and E_F position is 2.38 eV (**Figure S4d**) and the bandgap of $BiVO_4$ powder is 2.65 eV (**Figure S4b**), the E_{VB} of $BiVO_4$ powder is determined to

be -7.48 eV (vs. vacuum level). So, the E_{VB} and E_{CB} of $BiVO_4$ powder are determined to be 3.04 eV and 0.39 eV (vs. NHE). Therefore, the $BiVO_4$ powder has no ability to achieve overall water splitting. The relative position of energy band of the $BiVO_4$ powder and $BiVO_4$-FTO sample are shown in **Figure S4e,f**.

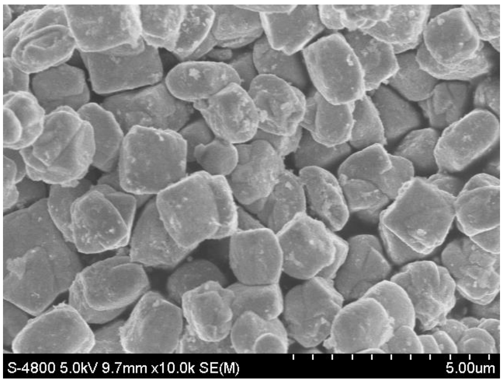

Figure S5 SEM image of tetragonal $BiVO_4$ powder synthesized by co-precipitation method.

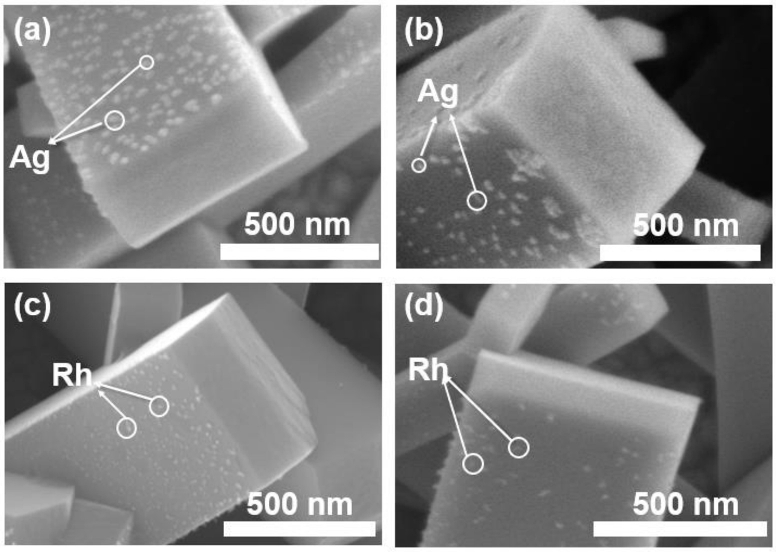

Figure S6 (a-b) SEM images of Ag-deposited $BiVO_4$. (c-d) SEM images of Rh-deposited $BiVO_4$.

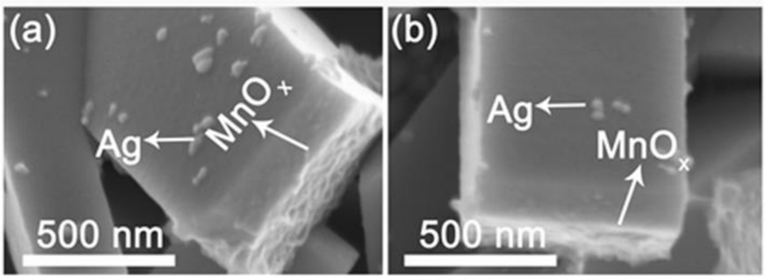

Figure S7 (a-b) SEM images of $BiVO_4$-FTO film after loading Ag and MnO_x.

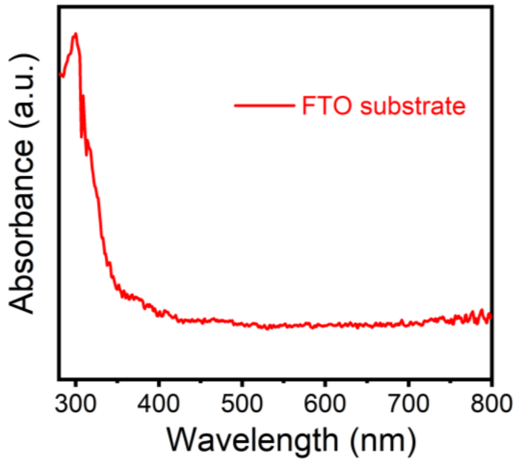

Figure S8 The diffuse reflection spectrum of FTO substrate.

The DRS of FTO substrate is shown in **Figure S8**. The absorption of FTO appears at 350 nm, which is basically the same as the light absorption of SnO_2 reported in the literature[5]. In order to eliminate the light absorption of the substrate inevitably contained in the collected samples, the visible light of $\lambda > 400$ nm was used to carry out the photocatalytic reaction in the experimental part.

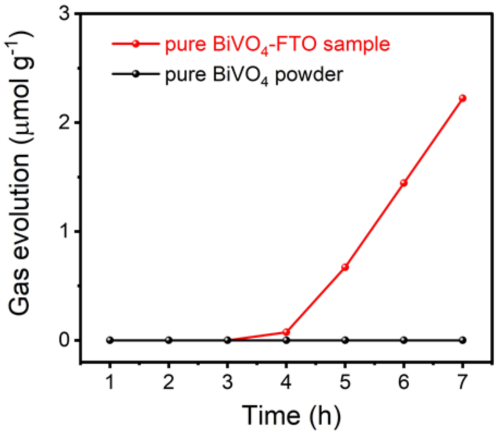

Figure S9 Photocatalytic overall water splitting reaction on $BiVO_4$-FTO sample and $BiVO_4$ powder without adding sacrificial agent and cocatalysts.

As shown in **Figure S9**, bare $BiVO_4$-FTO sample has no H_2 production activity during the first three hours of irradiation. From the third hour onwards, H_2 was slowly released, and O_2 was hardly detected due to the small release of O_2. By contrast, $BiVO_4$ powder had no gas escape even under long-term irradiation.

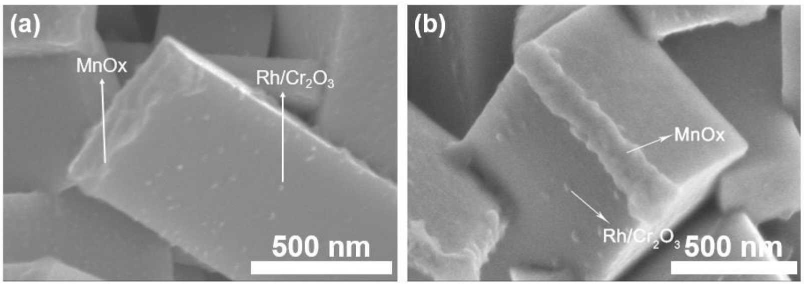

Figure S10 (a-b) SEM images of $BiVO_4$-FTO film after loading $Rh/Cr_2O_3/MnO_x$ cocatalysts.

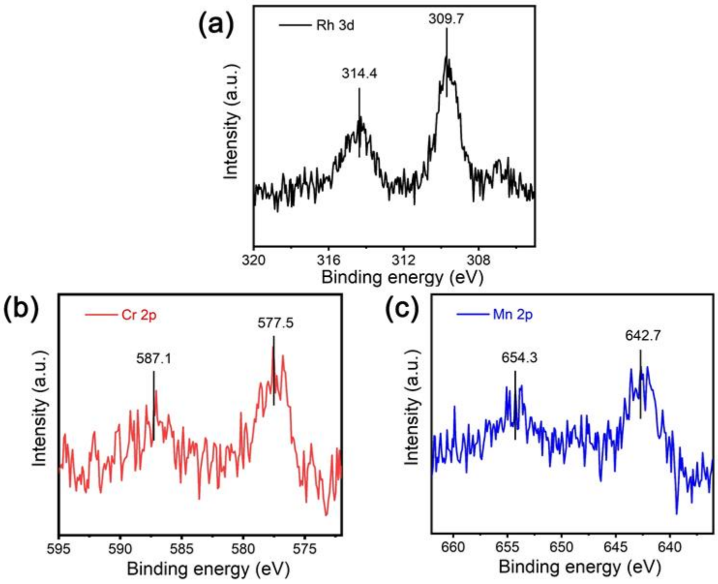

Figure S11 XPS spectra of (a) Rh 3d, (b) Cr 2p and (c) Mn 2p of cocatalysts $Rh/Cr_2O_3/MnO_x$. The Rh 3d spectrum shows two peaks at 314.4 eV and 309.7 eV, which are assigned to Rh^0. The peaks located at 587.1 eV and 577.5 eV are corresponding to Cr $2p_{1/2}$ and Cr $2p_{3/2}$, and the Mn 2p peaks at 654.3 and 642.7 eV are consistent with Mn $2p_{1/2}$ and Mn $2p_{3/2}$ orbitals.

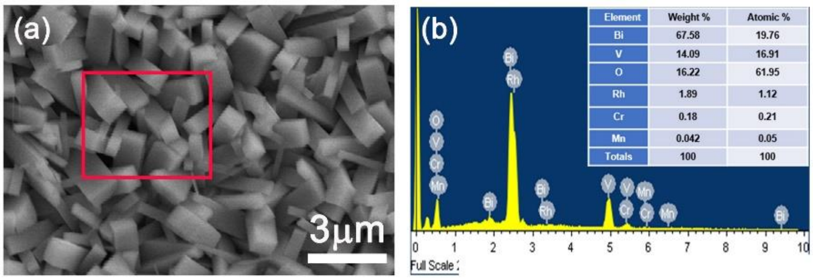

Figure S12 (a-b) Energy Dispersive Spectrometer (EDS) spectrum and the detailed information about the composition of $BiVO_4$-FTO sample after loading Rh/Cr_2O_3/MnO_x cocatalysts.

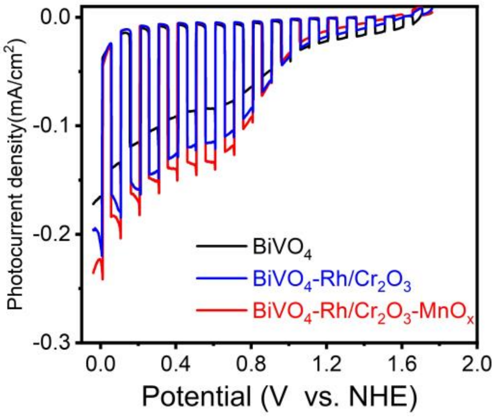

Figure S13 LSV analysis of pure $BiVO_4$, $BiVO_4$-Rh/Cr_2O_3 and $BiVO_4$-Rh/Cr_2O_3/MnO_x.

Figure S13 shows the photocurrent density-applied potential (*J*-*V*) curves of $BiVO_4$ before and after loading cocatalysts under chopped AM 1.5G sunlight. $BiVO_4$-Rh/Cr_2O_3/MnO_x yields a photocurrent density of about -0.22 mA cm^{-2} at 0 V vs NHE which is higher than pure $BiVO_4$ and single cocatalyst $BiVO_4$-Rh/Cr_2O_3.

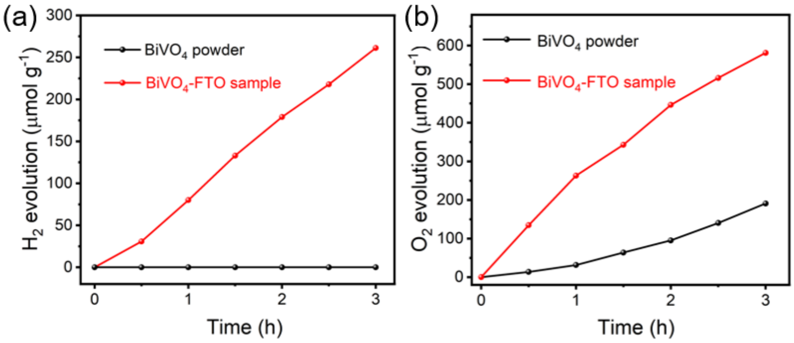

Figure S14 (a) Photocatalytic HER using hydrogen peroxide as hole sacrificial reagent. (b) Photocatalytic OER using $AgNO_3$ as electron sacrificial reagent of the $BiVO_4$ powder and $BiVO_4$-FTO sample after loading Rh/Cr_2O_3/MnO_x cocatalysts.

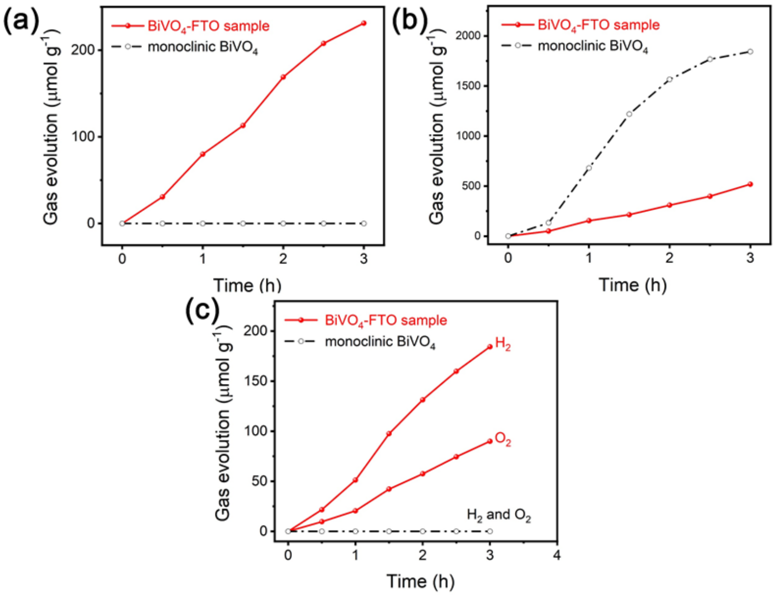

Figure S15 (a) Photocatalytic HER of $BiVO_4$-FTO sample and monoclinic $BiVO_4$ deposited with Rh/Cr_2O_3 under visible light (λ > 400nm) using hydrogen peroxide as hole sacrificial reagent. (b) Photocatalytic OER of $BiVO_4$-FTO sample and monoclinic $BiVO_4$ deposited with MnO_x under visible light (λ > 400nm) using $AgNO_3$ as electron sacrificial reagent. (c) Photocatalytic overall water splitting reaction of $BiVO_4$-FTO sample and monoclinic $BiVO_4$ deposited with $Rh/Cr_2O_3/MnO_x$ under visible light (λ > 400nm) without sacrificial agent.

The monoclinic $BiVO_4$ was prepared according to the previous literature.[6] As shown in **Figure S15a**, in the presence of sacrificial reagent, the monoclinic $BiVO_4$ cannot release hydrogen due to the limitation of CB position. However, the tetragonal $BiVO_4$ grown on FTO substrate can split water into H_2, which is related to the upward shift of CB after strain. As shown in **Figure S15b**, in the presence of sacrificial reagent, the oxygen released activity of monoclinic $BiVO_4$ is about 5 times that of tetragonal $BiVO_4$, which could attribute to the stronger absorption of visible light and fast oxidation reaction kinetics of monoclinic $BiVO_4$.[7] As depicted in **Figure S15c**, under the same loading conditions of cocatalysts, tetragonal $BiVO_4$ can overspilt water into H_2 and O_2 without adding sacrificial reagents, while monoclinic $BiVO_4$ does not show any activity.

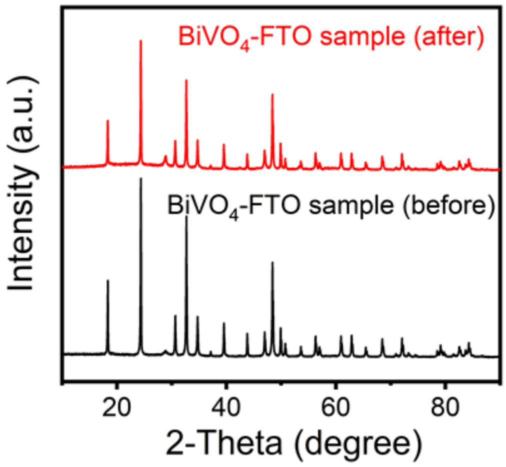

Figure S16 XRD patterns of as-prepared $Rh/Cr_2O_3/MnO_x$ loaded $BiVO_4$-FTO sample before and after 12 h photocatalytic overall water splitting reaction.

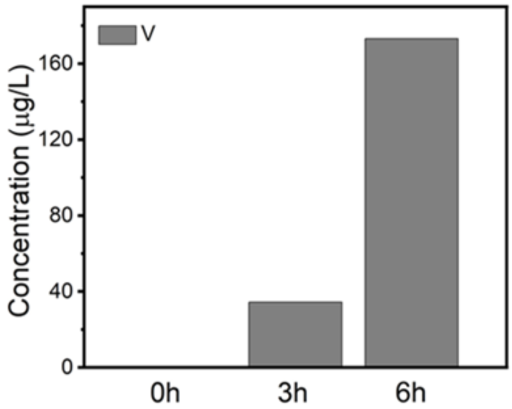

Figure S17 The ICP-OES analysis of vanadium concentrations in the pure water (pH ~ 7) after photocatalytic overall water splitting stability tests (0 h, 3 h and 6 h) for $Rh/Cr_2O_3/MnO_x$ modified $BiVO_4$-FTO sample.

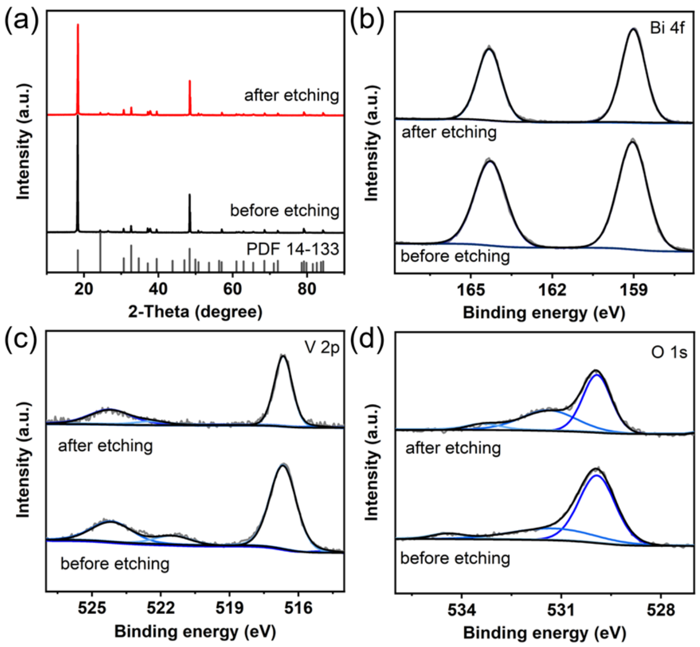

Figure S18 The XRD patterns (a) and XPS measurements (b-d) of $BiVO_4$-FTO film before and after being etched by alkali.

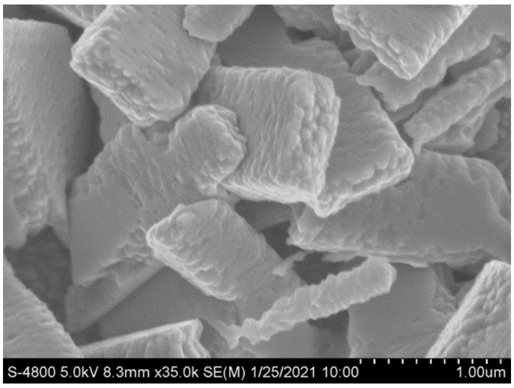

Figure S19 SEM image of $BiVO_4$-FTO film after being etched 6 hours by 2 M NaOH.

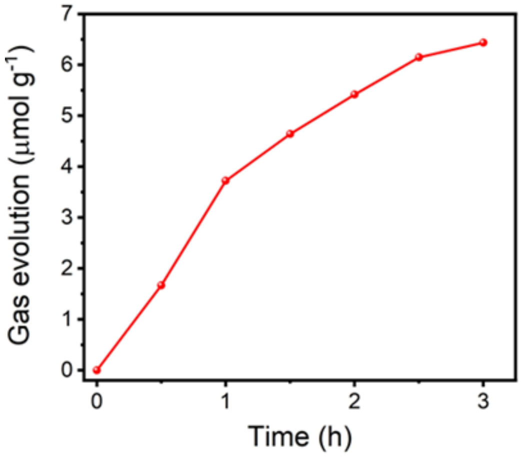

Figure S20 The photocatalytic activity of $BiVO_4$-FTO sample after crystal planes being etched.

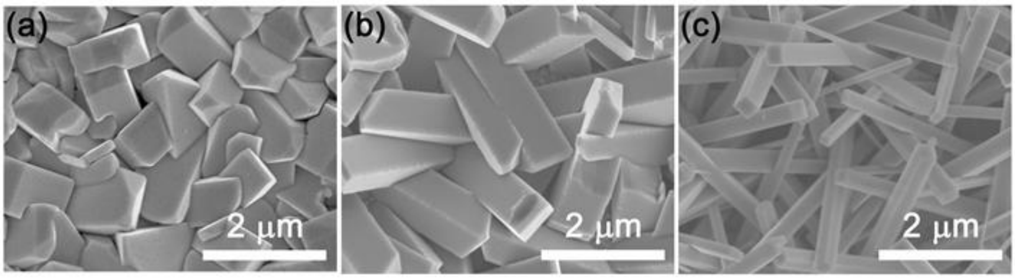

Figure S21 SEM images of (a) $BiVO_4$-FTO (pH=1.6), (b) $BiVO_4$-FTO (pH=2) and (c) $BiVO_4$-FTO (pH=3).

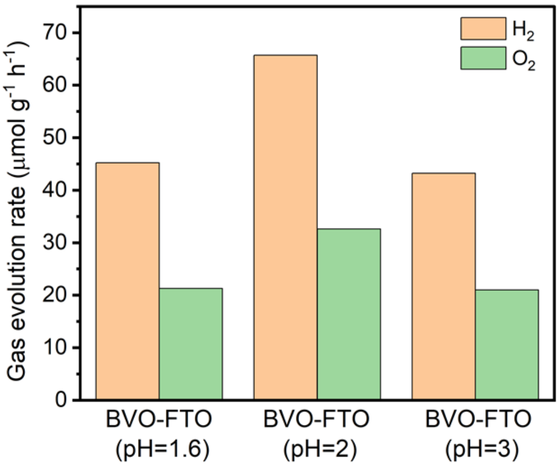

Figure S22 The gas generation rates of $BiVO_4$-FTO sample synthesized under different pH value.

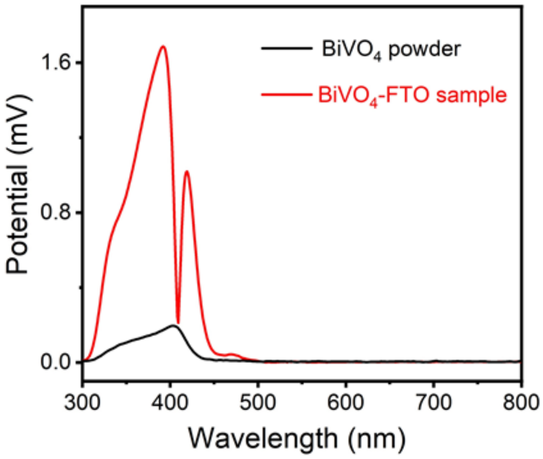

Figure S23 The surface photovoltage (SPV) spectra of $BiVO_4$ powder and $BiVO_4$-FTO sample.

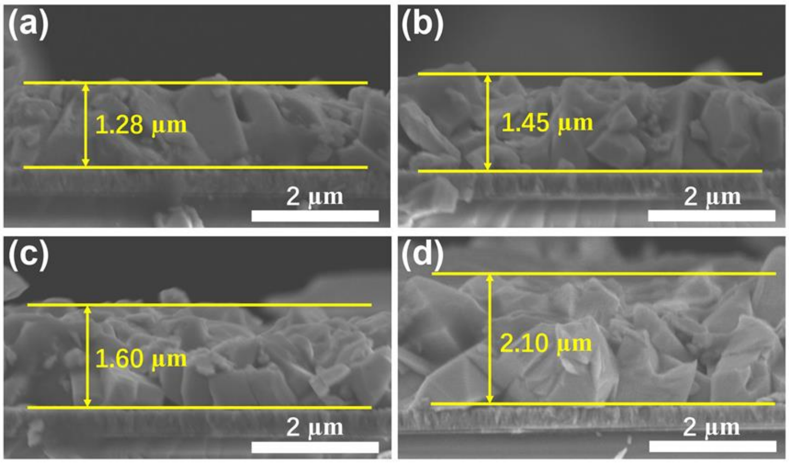

Figure S24 (a-d) SEM images of four different thicknesses of $BiVO_4$-FTO films synthesized under different hydrothermal time (3h, 9h, 12h, 24h).

$BiVO_4$ films with different thicknesses were synthesized under different hydrothermal time (3h, 9h, 12h, 24h), which shown in **Figure S24**. In this work, the cross-sectional SEM images of the $BiVO_4$-FTO films are shown in **Figure S24c** (12h), and the thickness is about 1.60 µm.

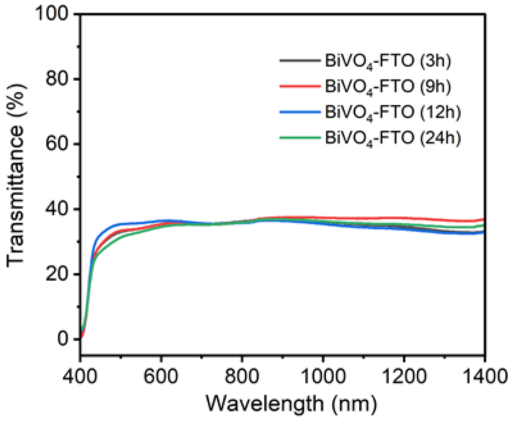

Figure S25 Optical transmittance spectra of $BiVO_4$-FTO films of different synthesis time.

It can be seen from **Figure S25** that the transmission has little dependence on the film thickness, and the light transmittance of films of all thicknesses is about 36%, indicating the thickness has

little relationship with the penetration depth of light, within the thickness range of this experiment.

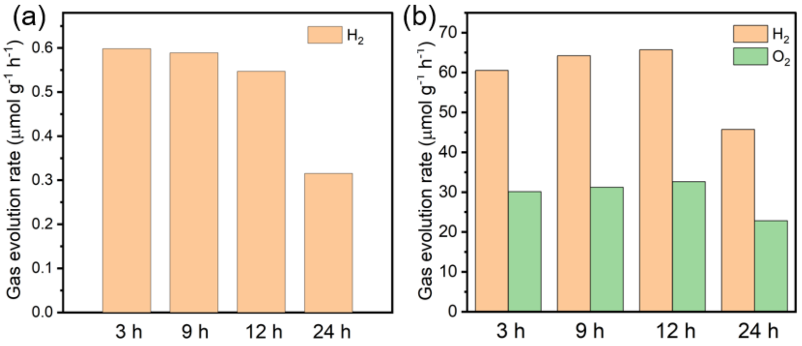

Figure S26 The gas generation rates of $BiVO_4$-FTO sample with different synthesis time (3h, 9h, 12h, 24h): (a) bare $BiVO_4$-FTO sample, (b) $BiVO_4$-FTO sample after loading cocatalysts.

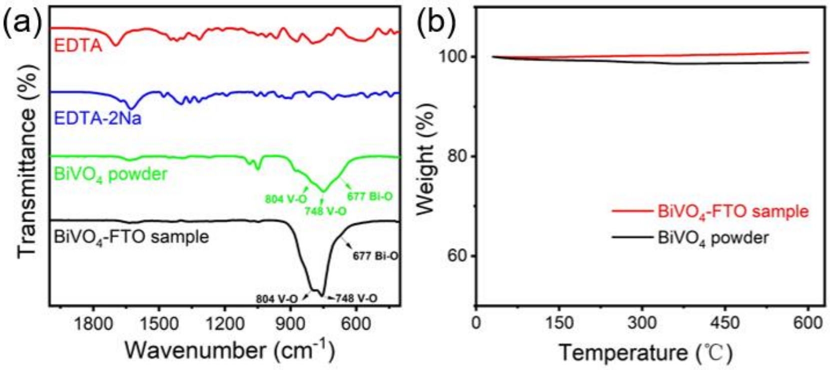

Figure S27 (a) Infrared spectra and (b) TG analysis of $BiVO_4$-FTO sample and $BiVO_4$ powder.

Table S1. Young's modulus E (GPa), Poisson ratio υ of tetragonal zircon $BiVO_4$.[8]

	E	υ
$BiVO_4$	111.00	0.33

Table S2. XRD refinement data of $BiVO_4$ powder, $BiVO_4$-FTO film (contain the substrate of SnO_2) and the $BiVO_4$-FTO sample collected from the substrate.

		$BiVO_4$ powder	$BiVO_4$-FTO film (containing SnO_2)	$BiVO_4$-FTO sample
Phase content		100	75.866	100
Cell parameters	a (Å)	7.3007	7.3044	7.3047
	b (Å)	7.3007	7.3044	7.3047
	c (Å)	6.4611	6.4623	6.4628
	α (°)	90	90	90
	β (°)	90	90	90
	γ (°)	90	90	90
Fitting index		Rwp=4.09 %	Rwp=8.856 %	Rwp=6.12 %

Table S3. Single-step photocatalysts for overall water splitting without using sacrificial reagents.

	H_2	O_2	Efficiency	Light source	Ref
$BaTiO_3$	159 µmol g^{-1} h^{-1}	75 µmol g^{-1} h^{-1}		300-W Xe lamp simulated sunlight (AM 1.5G)	*Angew. Chem. Int. Ed. 10.1002/anie.202103112*
MOF-derived $Bi_{0.5}Y_{0.5}VO_4$	124.2 µmol h^{-1}	61.7 µmol h^{-1}		300-W Xe lamp (λ > 300 nm)	*Chem. Eng. J. 2021, 15, 128911*
CdS	2.7 µmol h^{-1}	0.6 µmol h^{-1}	AQE: 0.32%, 0.23% and 0.10% at 365, 425 nm, and 475 nm respectively	300-W Xe lamp λ > 420 nm	*Adv. Sci. 2020, 7, 1903568*
NiCo hydroxide	34 µmol g^{-1} h^{-1}	16.8 µmol g^{-1} h^{-1}	AQE: 1.38% at 380 nm	simulated sunlight (AM 1.5G)	*Angew. Chem. Int. Ed. 2020, 28, 11607-11612*
$SrTiO_3$: Al	3.54 mmol h^{-1}	1.78 mmol h^{-1}	STH: 0.65% QE: 95.7%, 95.9% at 350, 360 nm, respectively	300-W Xe lamp full arc	*Nature 2020, 581, 386-388*
$Y_2Ti_2O_5S_2$	~2.50 µmol h^{-1}	~1.24 µmol h^{-1}	STH: 0.007%	300-W Xe lamp λ > 420 nm	*Nat. Mater. 2019, 18, 827-832*
CeO_2: Sm	~500 µmol h^{-1}	~250 µmol h^{-1}		450-W Hg lamp	*Chem. Lett. 2019, 48, 200-203*
Ta_3N_5	~10 µmol h^{-1}	~5 µmol h^{-1}	STH: 0.014%	300-W Xe lamp λ > 420 nm	*Nat. Catal. 2018, 1, 756-763*
p-GaN/InGaN	~6 mol g^{-1}	~3 mol g^{-1}	STH: 1.9%	simulated sunlight (AM 1.5G)	*J. Phys. Chem. C 2018, 122, 13797-13802*
g-C_3N_4	1.2 µmol h^{-1}	0.6 µmol h^{-1}	AQY: 0.3% at 405 nm	300-W Xe lamp λ > 420 nm	*Chem. Sci. 2016, 7, 3062-3066*
$LaMg_{1/3}Ta_{2/3}O_2N$	22 µmol h^{-1}	11 µmol h^{-1}	AQY: 0.18% at at 440 ± 30 nm	300-W Xe lamp (λ > 300 nm)	*Chem. - Eur. J. 2016, 22, 1854-1862*
$Bi_{1-x}In_xV_{1-x}Mo_xO_4$	~17 µmol h^{-1}	~8 µmol h^{-1}	AQY: 3.2% at 420-800 nm	450-W Hg lamp λ >420 nm	*Proc. Natl. Acad. Sci. 2015, 112, 13774-13778*
CoO	~11 mL	~5.5 mL	STH: 5%	0.9-W solid-state laser λ = 532 nm	*Nat. Nanotechnol. 2014, 9, 69-73*
Rutile TiO_2	3.4 µmol h^{-1}	1.6 µmol h^{-1}		300-W Xe lamp λ < 420 nm	*Chem. Commun. 2013, 49, 8404-8406*
$BiYWO_6$	11.2 µmol h^{-1}	5.8 µmol h^{-1}	AQY: 0.17% at 420 nm	500-W Xe lamp λ > 420 nm	*J. Phys. Chem. C. 2008, 112, 8521-8523*
$(Ga_{1-x}Zn_x)(N_{1-x}O_x)$	~463 µmol h^{-1}	~231 µmol h^{-1}	AQY: 2.5% at 420-440 nm	450-W Hg lamp λ > 400 nm	*Nature 2006, 440, 295; J. Am. Chem. Soc. 2012, 134, 8254-8259*
GaN: ZnO	~0.06 mmol h^{-1}	~0.03 mmol h^{-1}	AQE: 0.14% at 300-480 nm	450-W Hg lamp λ > 400 nm	*J. Am. Chem. Soc. 2005, 127, 8286-8287*
Tetragonal zircon $BiVO_4$	65.7 µmol g^{-1} h^{-1}	32.6 µmol g^{-1} h^{-1}	STH: 0.012% AQE: 0.046% at 365 nm	300-W Xe lamp λ > 400 nm	this work

References

[1] H. M. Zhang, J. B. Liu, H. Wang, W. X. Zhang, H. Yan, *Nanopart. Res.* **2008**, *10*, 767.

[2] X. Liang, P. Wang, F. Tong, X. Liu, C. Wang, M. Wang, Q. Zhang, Z. Wang, Y. Liu, Z. Zheng, Y. Dai, B. Huang, *Adv. Funct. Mater.* **2021**, *31*, 2008656.

[3] X. Bao, H. Li, Z. Wang, F. Tong, M. Liu, Z. Zheng, P. Wang, H. Cheng, Y. Liu, Y. Dai, Y. Fan, Z. Li, B. Huang, *Appl. Catal. B-Environ.* **2021**, *286*, 119885.

[4] X. Wu, J. Zhao, L. Wang, M. Han, M. Zhang, H. Wang, H. Huang, Y. Liu, Z. Kang, *Appl. Catal. B-Environ.* **2017**, *206*, 501.

[5] S. Sivakumar, E. Manikandan, *J. Mater. Sci. Mater. Electron.* **2019**, *30*, 7606.

[6] R. Li, F. Zhang, D. Wang, J. Yang, M. Li, J. Zhu, X. Zhou, H. Han, C. Li, *Nat. Commun.* **2013**, *4*, 1432.

[7] Y. Park, K. J. Mcdonald, K. Choi, *Chem. Soc. Rev.* **2013**, *42*, 2321.

[8] A. K. M. F. Islam, M. N. H. Liton, H. M. T. Islam, M. A. Helal, M. Kamruzzaman, *Chin. Phys. B.* **2017**, *26*, 395.
