# Supplementary material for: Strain Adjustment Realizes the Photocatalytic Overall Water Splitting on Tetragonal Zircon BiVO4
Source: Adv Sci (Weinh). 2022 Mar 22;9(15):2105299. doi: 10.1002/advs.202105299 (PMC9131610; doi:10.1002/advs.202105299)
Supplement: Supplementary file 2 — Supporting Information [file ADVS-9-2105299-s001.pdf]

ADVANCED SCIENCE
Open Access

Supporting Information

for *Adv. Sci.*, DOI 10.1002/advs.202105299

Strain Adjustment Realizes the Photocatalytic Overall Water Splitting on Tetragonal Zircon $BiVO_4$

*Dujuan Dai, Xizhuang Liang, Beibei Zhang, Yuanyuan Wang, Qian Wu, Xiaolei Bao, Zeyan Wang, Zhaoke Zheng, Hefeng Cheng, Ying Dai, Baibiao Huang and Peng Wang**

New Project report

Experimental data

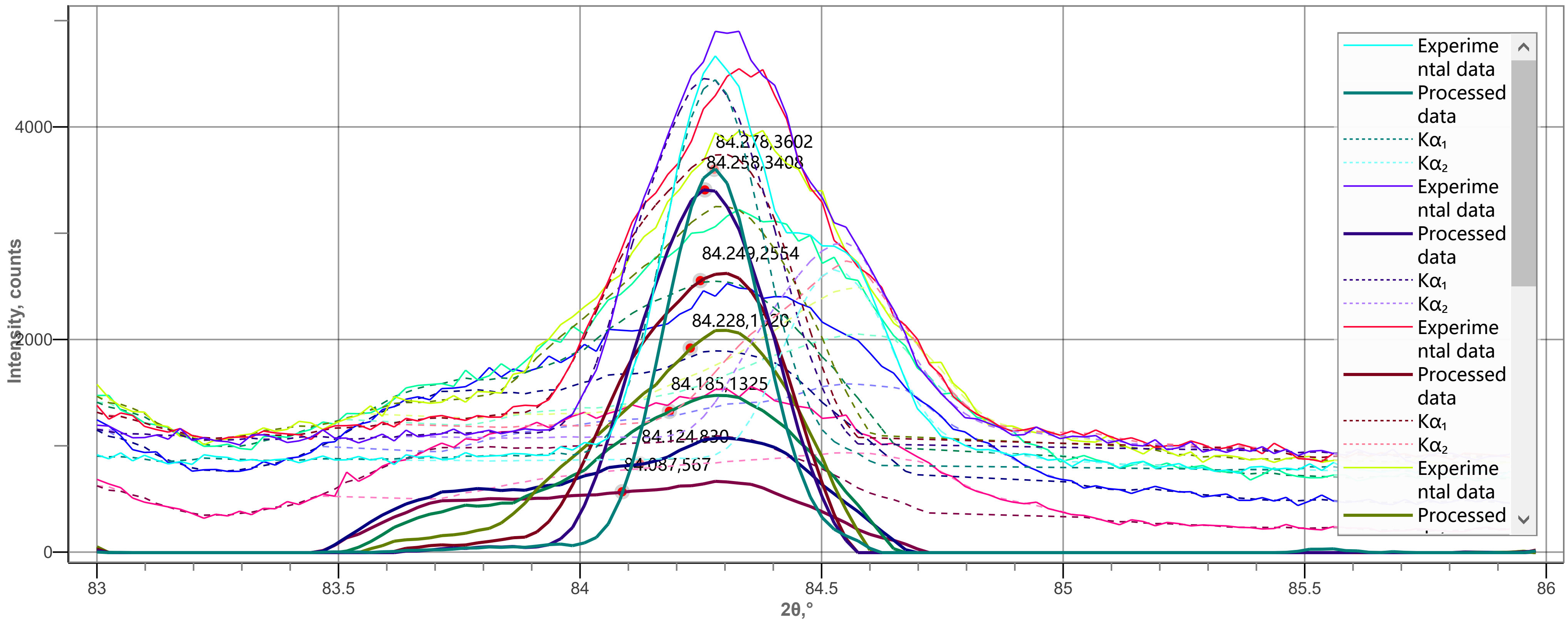

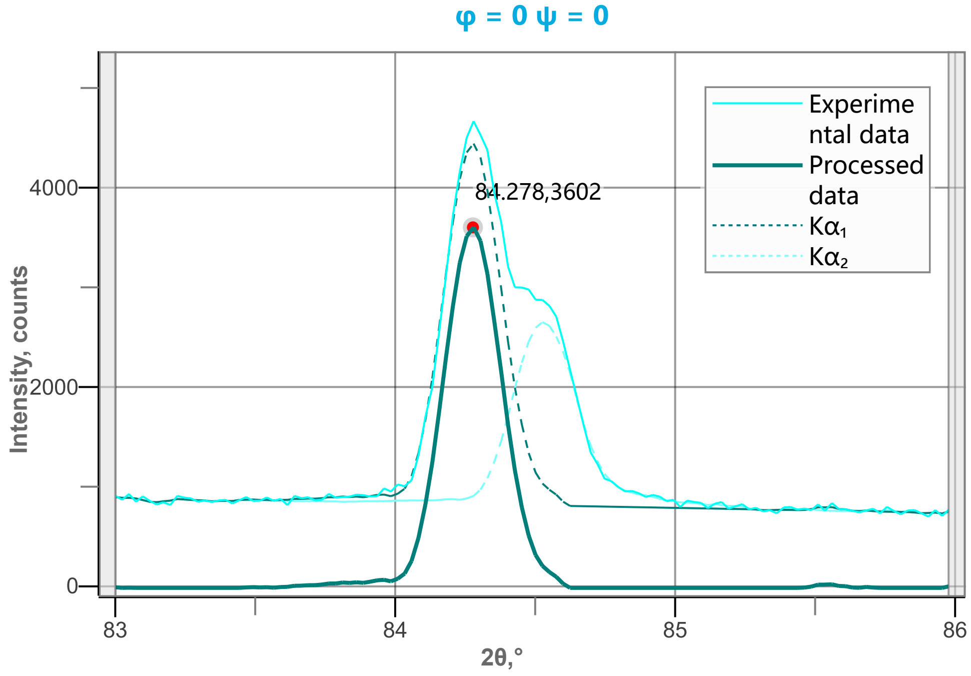
φ = 0 ψ = 0
Experimental data
Processed data
Kα1
Kα2
84.278,3602
Intensity, counts
0
2000
4000
83
84
85
86
2θ,°

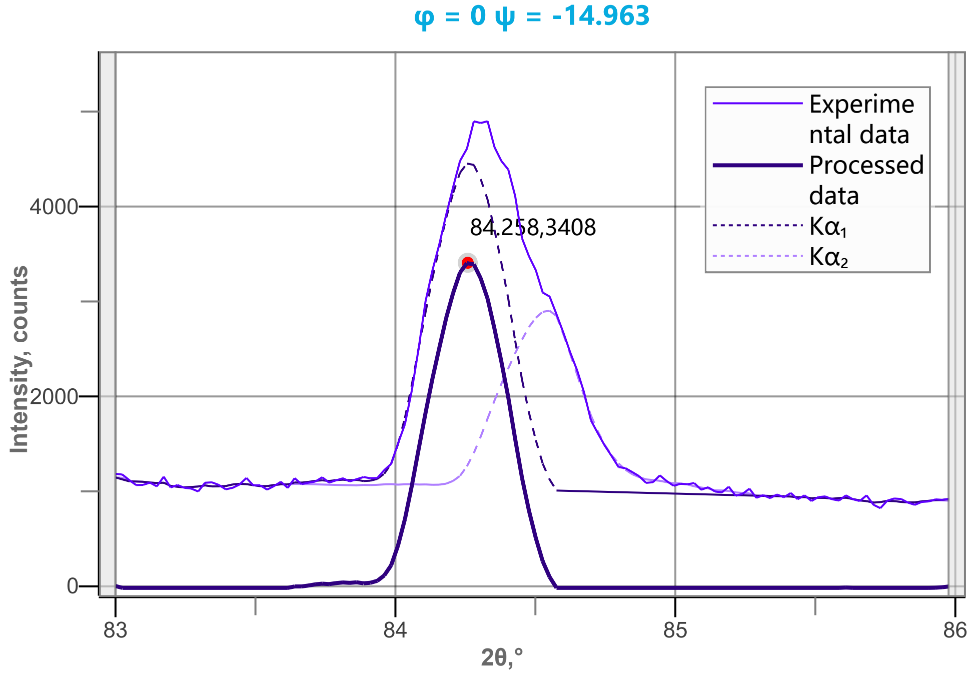
φ = 0 ψ = -14.963
Experimental data
Processed data
Kα1
Kα2
84.258,3408
Intensity, counts
0
2000
4000
83
84
85
86
2θ,°

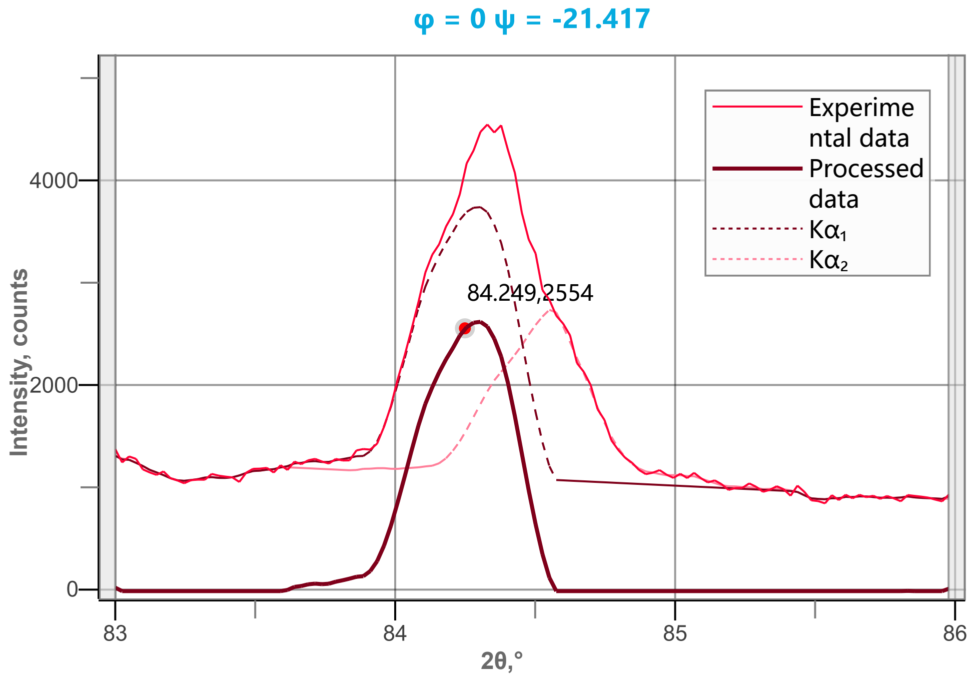
φ = 0 ψ = -21.417
Experimental data
Processed data
Kα1
Kα2
84.249,2554
Intensity, counts
0
2000
4000
83
84
85
86
2θ,°

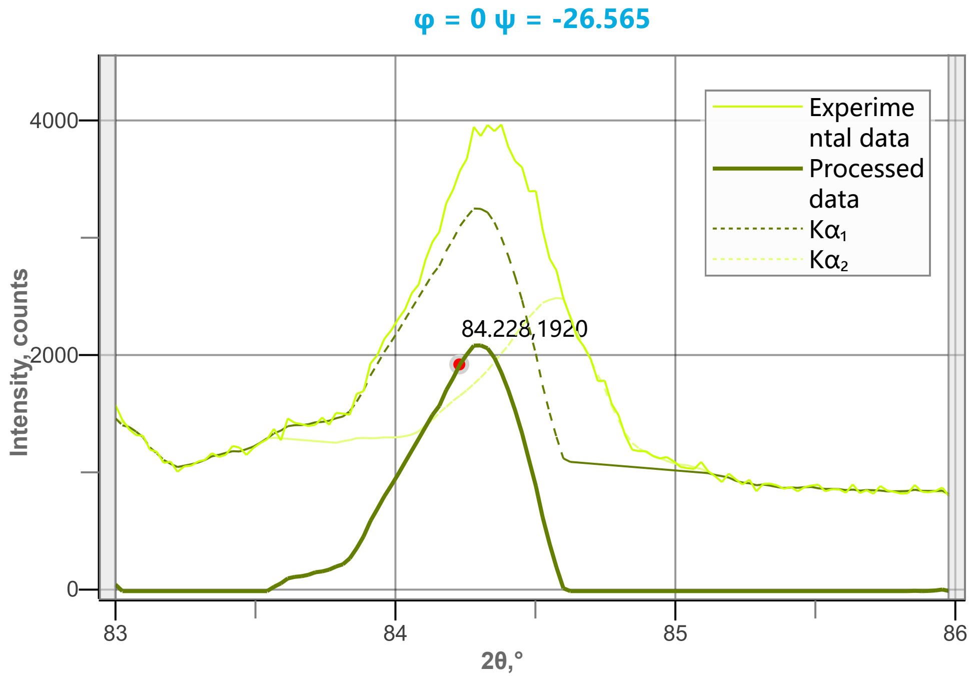
φ = 0 ψ = -26.565
Experimental data
Processed data
Kα1
Kα2
84.228,1920
Intensity, counts
0
2000
4000
83
84
85
86
2θ,°

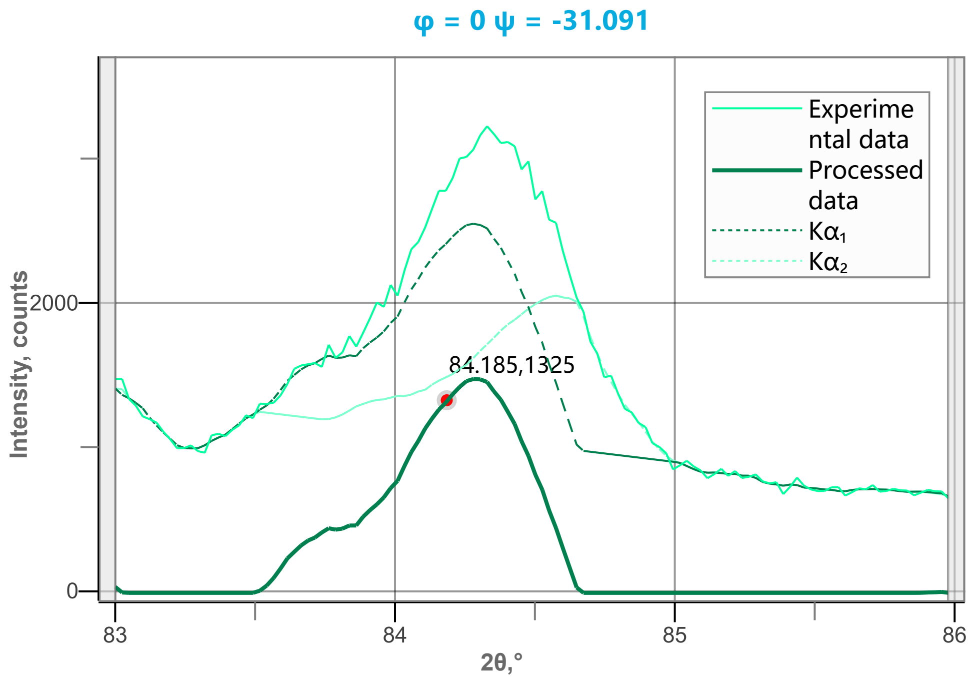
φ = 0 ψ = -31.091
Experimental data
Processed data
Kα1
Kα2
84.185,1325
Intensity, counts
2000
0
83
84
85
86
2θ,°

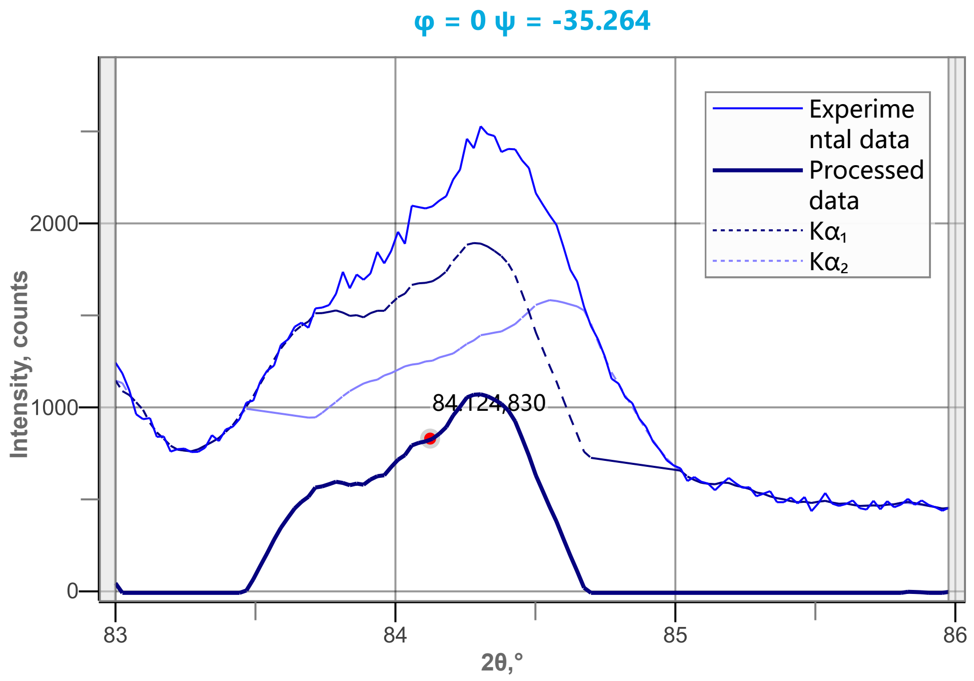
φ = 0 ψ = -35.264
Experimental data
Processed data
Kα1
Kα2
84.124,830
Intensity, counts
2000
1000
0
83
84
85
86
2θ,°

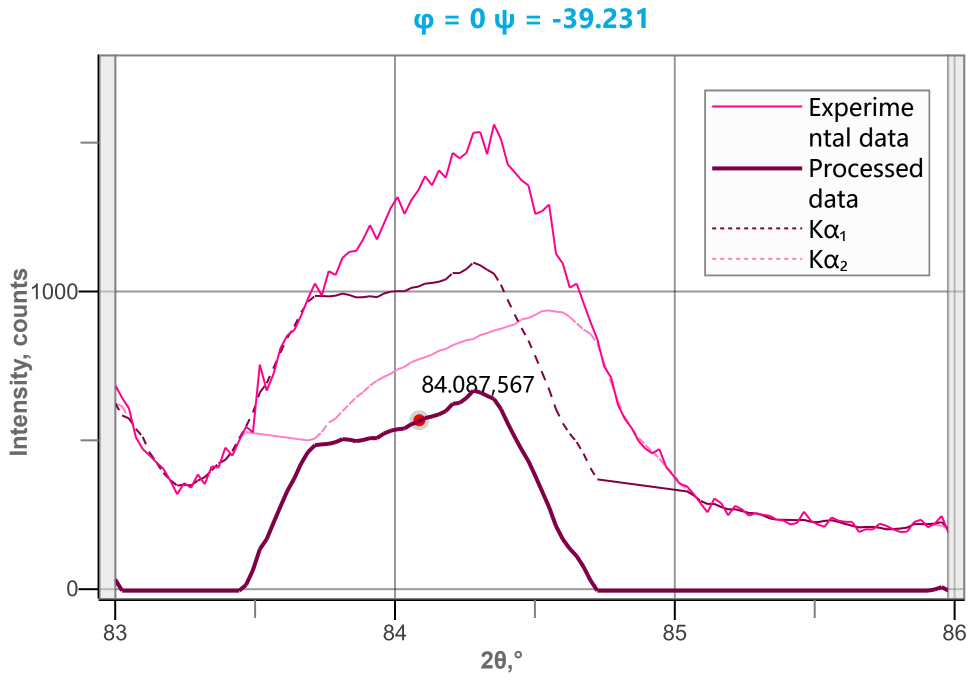
φ = 0 ψ = -39.231
Experimental data
Processed data
Kα1
Kα2
84.087,567
Intensity, counts
1000
0
83
84
85
86
2θ,°

Sample Model

		Material				
	⊟	Dreyerite, syr				
		hkl	Young's Modulus (E), MPa	Poisson Ratio (ν)	XEC Source	$2\theta_0$,°
		4 2 4 Custor	111000.0	0.3300	Manual	84.200

Data Processing

Corrections setup

Smoothing
- Method: Weighted average
- Points: 5

Background
- Model: Line connecting both end points
- Avg. points left: 5 Avg. points right: 5

$K\alpha_2$
- Fraction: 0.497
- Wavelength $K\alpha_1$, nm:: 0.154059292
- Wavelength $K\alpha_2$, nm:: 0.1544414

Peak detection setup

Search method: Center of gravity
Min reliability: 3

Peak List

#	Used	φ Group Value	2θ,°	Width,°	Integral Breadth,°	Intensity, counts	Integrated Intensity, counts	Initial
1	☑	180.000	84.277	[illegible]	0.236	3601	922	0.0[illegible]
2	☑	180.000	84.258	0.321	0.325	3407	1108	0.0[illegible]
3	☑	180.000	84.248	0.400	0.413	2553	1055	0.0[illegible]
4	☑	180.000	84.228	0.465	0.514	1919	986	0.0[illegible]
5	☑	180.000	84.184	0.531	0.654	1324	865	0.0[illegible]
6	☑	180.000	84.123	1.026	0.926	830	769	0.0[illegible]
7	☑	180.000	84.087	0.975	0.956	567	542	0.0[illegible]

$\sin^2\psi$ Plot (φ = 180)

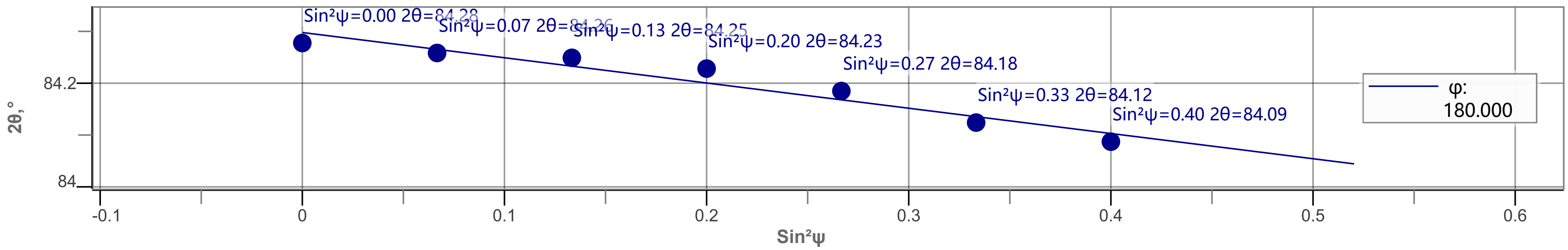

Results

Stress tensor

$$\sigma_{ij} = \begin{bmatrix} 390.29 \pm 47.44 & - & - \\ - & - & - \\ - & - & - \end{bmatrix} \text{MPa}$$

Other

2θ at ψ=0,°: 84.298 ± 0.014

σ_{vm}, MPa: 390.29
